# Medical and Surgical Treatment of Reproductive Outcomes in Polycystic Ovary Syndrome: An Overview of Systematic Reviews

**DOI:** 10.22074/ijfs.2020.5608

**Published:** 2019-11-11

**Authors:** Moustafa A. Gadalla, Robert J. Norman, Chau T Tay, Danielle S. Hiam, Angela Melder, Jyotsna Pundir, Shakila Thangaratinam, Helena J Teede, Ben W. J. Mol, Lisa J. Moran

**Affiliations:** 1 Women’s Health Hospital, Department of Obstetrics and Gynaecology, Assiut University, Assiut, Egypt; 2 Robinson Research Institute, Discipline of Obstetrics and Gynaecology, University of Adelaide, Adelaide, Australia; 3 Monash Centre for Health Research and Implementation, School of Public Health and Preventive Medicine, Victoria, Melbourne, Australia; 4 Monash Diabetes and Endocrinology Units, Monash Health, Victoria, Melbourne, Australia; 5 Institute of Sport, Exercise and Active Living, Victoria University, Melbourne, Australia; 6 Centre of Reproductive Medicine, St Bartholomew’s Hospital, London, United Kingdom; 7 Women’s Health Research Unit, Barts and the London School of Medicine and Dentistry, Queen Mary University London, London, United Kingdom; 8 Department of Obstetrics and Gynaecology, Monash University, Clayton, Victoria, Melbourne, Australia

**Keywords:** Infertility, Polycystic Ovary Syndrome, Review, Therapeutics, Treatment Outcome

## Abstract

Polycystic ovary syndrome (PCOS) is a common, complex condition that affects up to 18% of reproductive- aged women, causing reproductive, metabolic and psychological dysfunctions. We performed an overview and appraisal of methodological quality of systematic reviews that assessed medical and surgical treatments for reproductive outcomes in women with PCOS. Databases (MEDLINE, EMBASE, CINAHL PLUS and PROSPERO) were searched on the 15th of September 2017. We included any systematic review that assessed the effect of medical or surgical management of PCOS on reproductive, pregnancy and neonatal outcomes. Eligibility assessment, data extraction and quality assessment by the Assessing the Methodological Quality of Systematic Reviews (AMSTAR) tool were performed in duplicate. We identified 53 reviews comprising 44 reviews included in this overview; the majority were moderate to high quality. In unselected women with PCOS, letrozole was associated with a higher live birth rate than clomiphene citrate (CC), while CC was better than metformin or placebo. In women with CC-resistant PCOS, gonadotrophins were associated with a higher live birth rate than CC plus metformin, which was better than laparoscopic ovarian drilling (LOD). LOD was associated with lower multiple pregnancy rates than other medical treatments. In women with PCOS undergo- ing in vitro fertilization/intracytoplasmic sperm injection (IVF/ICSI), the addition of metformin to gonadotro- phins resulted in less ovarian hyperstimulation syndrome (OHSS), and higher pregnancy and live birth rates than gonadotrophins alone. Gonadotrophin releasing hormone (GnRH) antagonist was associated with less OHSS, gonadotrophin units and shorter stimulation length than GnRH agonist. Letrozole appears to be a good first line treatment and gonadotrophins, as a second line treatment, for anovulatory women with PCOS. LOD results in lower multiple pregnancy rates. However, due to the heterogeneous nature of the included popula- tions of women with PCOS, further larger scale trials are needed with more precise assessment of treatments according to heterogeneous variants of PCOS.

## Introduction

Polycystic ovary syndrome (PCOS) is one of the most important dilemmas in reproductive medicine. PCOS is a member of the World Health Organization group II ovulation disorders, and has a 9-18% prevalence among reproductive-aged women (1) and nearly 80% among infertile anovulatory women (1, 2). There is an ongoing debate related to its definition, aetiology, diagnosis and treatment for its clinical phenotypes (3). Since first described by Stein and Leventhal (4), a number of reports and meetings have suggested diagnostic criteria for this condition (3, 5, 6). However, the criteria reported by ESHRE/ASRM in Rotterdam in 2003 are most commonly used both in research and clinical care. These criteria propose that two out of three domains should be present to establish a diagnosis of PCOS. These domains are: an-/oligo-ovulation, hyperandrogenism (clinical ± biochemical) and polycystic ovary morphology on ultrasound examination, with exclusion of other causes of hyperandrogenism (6). In 2012, the National Institute of Health reinforced the need for identification of four phenotypes within the Rotterdam criteria in women with PCOS, which refer to the combination of diagnostic criteria (7). By using the possible combinations of these criteria, four different phenotypes of PCOS are now identified: i. Hyperandrogenism (clinical or biochemical) and chronic anovulation (H-CA), ii. Hyperandrogenism and polycystic ovaries on ultrasound (PCOm), but with ovulatory cycles (H-PCOm), iii. Chronic anovulation and polycystic ovaries without hyperandrogenism (CA-PCOm), and iv. Hyperandrogenism, chronic anovulation and polycystic ovaries (H-CA-PCOm). The identification of specific phenotypes in women with PCOS seems to be justified from the metabolic point (3). 

This heterogeneous condition manifests with many clinical presentations, including infertility, pregnancy complications, and psychological and metabolic features. The reproductive problems associated with PCOS consist mainly of menstrual dysfunction, infertility and pregnancy complications. Many treatments are proposed by different guidelines for infertility with PCOS, and include clomiphene citrate (CC), letrozole and gonadotrophins. However, there is a lack of clarity around the relative efficacy of these different treatments. Despite the agreement between most guidelines of the importance and priority of lifestyle modification in PCOS and weight loss, where women are overweight or obese, there are still limited studies that compare lifestyle modification and pharmacological drugs for reproductive outcomes (8). With regards to pharmacological treatment in isolation, CC is recommended as first-line treatment for ovulation induction (OI) in infertile women with POCS with the alternative treatment, letrozole, which has encouraging results in many recent trials (1, 2, 8-10). Although the insulin sensitizer metformin has been recently recommended as a firstline treatment (11), its role and specific indication are controversial (1-3). The second-line treatment is usually recommended as gonadotrophins or laparoscopic ovarian drilling (LOD) (2). Additional issues relating to treatment of reproductive outcomes which are still somewhat controversial include the best time to use *in vitro* fertilization/intracytoplasmic sperm injection (IVF/ICSI) in women who failed to become pregnant after pharmacological treatment, and the potential benefit of modern techniques like in vitro maturation (IVM) (2, 3).

The aim of this review was to perform an overview to summarize and appraise the content, results and quality of systematic reviews that assess medical or surgical treatments for reproductive outcomes in women with PCOS.

## Materials and Methods

### Inclusion criteria

The Participant, Intervention, Comparison, Outcomes and Studies (PICOS) framework was used for this review. This overview is part of a larger overview of systematic reviews. For this broader overview, we included any systematic review or meta-analysis where the assessment or management of PCOS was the primary focus of the review, either as interventions in PCOS or a comparison of women with and without PCOS for a specific outcome. Exclusion criteria were studies where PCOS was a secondary condition assessed as part of a broader topic. For this specific overview, we included any systematic review that assessed the effect of medical or surgical management of PCOS on reproductive outcomes. The specific inclusion criteria were: i. Published from 2009 onwards, as this was the date of publication of the Preferred Reporting Items for Systematic Reviews and Meta-Analyses (PRISMA) statement as a guideline for conducting systematic reviews (12), ii. Must have included a search strategy that contained at least key words or terms, iii. Must include the number of identified and included articles, and iv. The review needed to conduct some form of quality appraisal of the articles. The comparisons term was not applicable in this review context. The outcomes assessed were reproductive outcomes, specifically live birth, clinical pregnancy, miscarriage, ovulation, multiple pregnancy, menstrual cycle frequency, follicular size, pregnancy related outcomes (gestational diabetes, pregnancy-induced hypertension and pre-eclampsia), neonatal outcomes, costs and side effects. Only articles published in English were included. The protocol is registered in the International Prospective Register of Systematic Reviews PROSPERO (CRD42016052649).

### Article selection

A comprehensive database search was conducted on the 17^th^ of October 2016, which was last updated on 15^th^ September 2017. The following electronic databases were used to identify relevant published literature: Medline in-process and other non-indexed citations [Ovid MEDLINE(R) In-Process & Other Non-Indexed Citations, Ovid MEDLINE(R) Daily and Ovid MEDLINE(R) 1946 to Present]; EMBASE (EBM Reviews- Cochrane Database of Systematic Reviews 2005 to September 15, 2017, EBM Reviews- ACP Journal Club 1991 to September 2017, EBM Reviews- Database of Abstracts of Reviews of Effects 1^st^ Quarter 2016, EBM Reviews- Cochrane Central Register of Controlled Trials September 2017, EBM Reviews- Cochrane Methodology Register 3^rd^ Quarter 2012, EBM Reviews- Health Technology Assessment 4^th^ Quarter 2016, EBM Reviews- NHS Economic Evaluation Database 1^st^ Quarter 2016); and CINAHL PLUS. The search strategy is documented in Supplementary Appendix 1 (See Supplementary On line Information at www.celljournal.org). This search was modified for EMBASE and CINAHL using their subject headings instead of the MeSH subject headings. The International Prospective Register of Systematic Reviews PROSPERO (http://www.crd.york.ac.uk/PROSPERO/) was additionally searched on the 15^th^ September 2017 using the key words “PCOS” or “polycystic ovary syndrome”. In addition, experts in the field were asked to provide any potentially relevant studies for consideration. Two independent reviewers, who were not blinded to the names of investigators or sources of publication, identified and selected the articles that met the inclusion criteria (L.J.M, D.H or C.T.T). Disagreements between reviewers were discussed and resolved by consensus or arbitration with a third reviewer.

### Data extraction

All eligible systematic reviews included were examined and extracted independently by two reviewers (L.J.M, M.G or C.T.T). Disagreements were discussed and resolved by consensus or arbitration with a third reviewer. The data extracted included information on author(s), year, country of author, inclusion criteria, study methodology, study outcomes, number of studies identified, number of participants in the review, whether a meta-analysis was conducted, and quality of identified articles in each review (as reported by the systematic review authors as overall quality of the entire study or evidence or reported as unclear if not summarized by the systematic review authors).

### Data synthesis

 A narrative description of the included reviews was performed. We presented results per reproductive outcome.

### Quality assessment of systematic reviews

All included reviews were evaluated by two independent reviewers (L.J.M, M.G or C.T.T) using the Assessing the Methodological Quality of Systematic Reviews (AMSTAR) tool (13, 14). Disagreements were discussed and resolved by consensus or arbitration with a third reviewer. The AMSTAR tool contains 11 items to appraise the methodological aspects of the systematic reviews. Each item was scored 1 for “yes” and 0 for “no” or “not applicable” with a total score range from 0 to 11. The methodological quality for each review was classified as low [≤ 3], moderate [4-7] and high [8-11] (15) .

## Results

### Characteristics of included reviews

**Fig 1:**
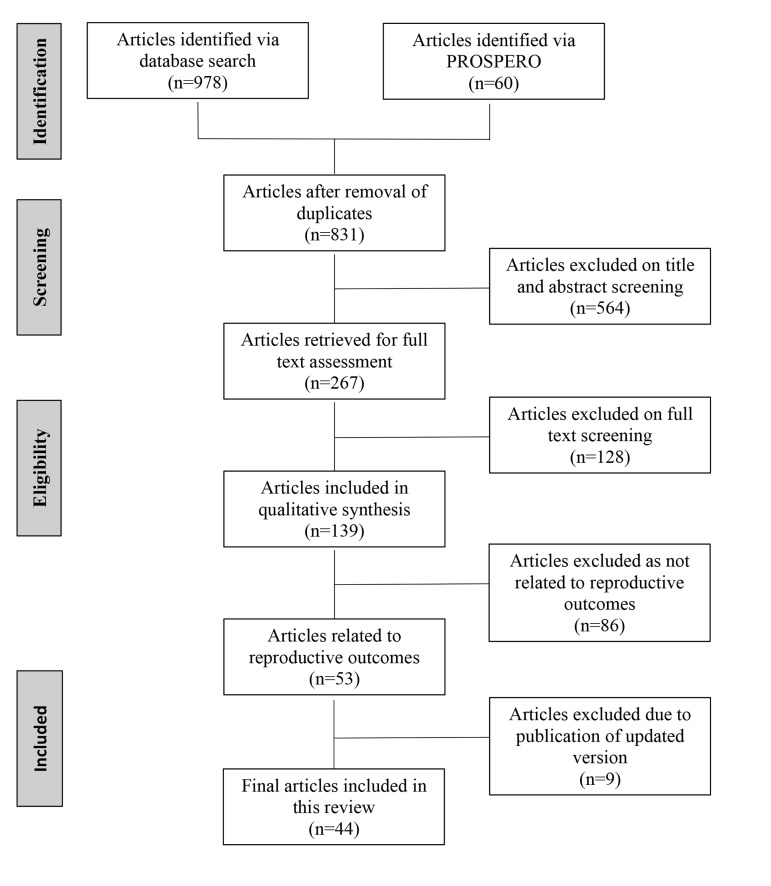
Study selection.

The search yielded 978 citations, with 60 citations identified from PROSPERO and one citation identified from expert assessors, for a total of 1039 citations. There were 831 citations that remained after removal of duplicates. Based on a priori selection criteria, screening for title or abstract identified 276 articles for assessment of the full text. Of these, 128 articles were excluded for the following: not conducting quality assessment, not in English, no search terms detailed or no identified search strategy (Supplementary Appendix 2) (See Supplementary Online Information at www.celljournal.org). We included 139 full-text articles for our final analysis, of which 53 articles were related to the theme of medical or surgical treatment on reproductive outcomes in PCOS, with the remaining eligible articles assessed in separate overviews of systematic reviews and excluded from this specific review. These 53 articles comprised 44 reviews (Fig .1).

The characteristics of these reviews are summarized in Supplementary Appendix 3 (See Supplementary Online Information at www.celljournal.org). The number of included studies in each review ranged between none (16, 17) and 66 (18). The type of included studies in each review was only randomised controlled trials (RCTs) in 22 reviews (16, 18-38), RCTs and crossover trials until first inclusion in 11 reviews (17, 33, 39-47), RCTs and systematic reviews of RCTs in two reviews (48, 49), any study design in two reviews (50, 51), any study with control group in three reviews (52-54), RCTs and prospective studies in one review (55) and not stated in three reviews (56-58). Participants in the included reviews were treatment-naive women in two reviews (27, 28), women resistant to CC in six reviews (19, 23, 29, 32, 33, 56), women whose treatment status was undefined in 32 reviews (16-18, 20-22, 24-26, 30, 31, 35-42, 44-51, 55, 57, 59), pregnant women with PCOS in four reviews (52-54, 58), adolescents with PCOS (11-19 years old) in one review (34), and women with PCOS not trying to conceive in one review (43). Twenty-two reviews were conducted according to prior guidelines for conducting systematic reviews such as PRISMA, Meta-analyses Of Observational Studies in Epidemiology (MOOSE), Quality of Reporting of Meta-analyses (QUORUMS) or Cochrane (16, 17, 19, 23, 25, 27, 29, 32, 34, 36, 39-46, 50, 51, 58, 59). Meta-analyses were performed in 39 reviews (18-32, 34- 43, 45-50, 52-59). The systematic reviews did not apply language restrictions in 28 reviews (16, 17, 19, 20, 24-26, 28, 29, 34, 36, 38-47, 50, 53-56, 58, 59), restricted the search to articles in English in 12 reviews (18, 21, 22, 27, 32, 33, 35, 37, 48, 49, 52, 57), restricted the search to articles in English and Chinese in two reviews (30, 31) and did not state if language restrictions were applied in two reviews (23, 51). The quality of included studies in each review was not reported by authors or was not able to be easily interpreted in 31 reviews (16, 17, 20-29, 32, 36, 38, 39, 42, 43, 45-47, 50-59), low or insufficient in eight reviews (18, 31, 34, 35, 37, 40, 41, 44), low to moderate in two reviews (19, 48) and low to high in three reviews (30, 33, 49) 

### Quality of included reviews

The quality of the included reviews are presented in Supplementary Appendix 4 (See Supplementary Online Information at www.celljournal.org). Seven reviews were of low quality (28, 30, 33, 36, 51, 52, 58), 22 reviews were of moderate quality (16, 20-27, 31, 32, 35, 37, 38, 40, 50, 53-57, 59) and 15 reviews were of high quality (17-19, 29, 34, 39, 41-49). Twenty reviews had pre-specified their clinical question and inclusion criteria (16-19, 29, 33, 34, 39-49, 55, 59). Nineteen reviews conducted study selection and data extraction in duplicate (17-19, 21, 23, 26, 27, 29, 32, 34, 37, 39, 42-45, 50, 55, 57). Twenty-eight reviews conducted a comprehensive literature search (16-19, 21, 24-26, 28- 31, 34, 38-49, 53, 54, 59). Twenty reviews included grey literature searches (16, 17, 19, 25, 26, 29, 34, 38- 47, 53, 54, 59). Twenty-four reviews listed included and excluded studies (16, 17, 19, 23-27, 29, 32, 34, 38, 39, 41-46, 48-50, 57, 59). Forty reviews described the characteristics of the included studies (18-29, 32-59). Thirty-eight reviews assessed study quality (16-27, 29- 35, 37-50, 54, 56, 57, 59, 60). Nineteen reviews used the scientific quality of their included studies in formulating results (18, 20-22, 24, 25, 29, 31, 32, 34, 35, 39, 40, 45-49, 57). Thirty-seven reviews combined the studies using appropriate methods (18-32, 35-43, 45, 46, 48-50, 52-59). Twenty-two reviews addressed the risk of reporting bias, and used a statistical test where appropriate (16-19, 32, 34, 35, 37-39, 41-44, 46, 47, 50, 52, 53, 55, 56, 58). Seven reviews addressed the potential for conflict of interest (16, 17, 29, 43, 47-49).

### Types of interventions

#### Letrozole

Six reviews (three high quality (19, 39, 49) and three moderate quality (20, 27, 32) assessed interventions that contained letrozole, comprising a total of 89 trials and 14 008 participants. Of these, five assessed letrozole ± other OI drugs versus OI drugs, including letrozole alone (20, 27, 32, 39, 49) and one assessed letrozole versus LOD (19). The populations studied were women with PCOS who were treatment-naïve (27), CC resistant (32), or treatment-naïve ± CC resistant or unknown treatment status (20, 39, 49). 

The meta-analyses reported statistically significant results for higher live birth, pregnancy and ovulation after letrozole compared to CC followed by timed intercourse in overall women with PCOS, and higher live birth and pregnancy after letrozole in women with PCOS and body mass index (BMI) >25 kg/m^2^ (20, 27, 39, 49). In women with CC resistance, letrozole with or without metformin resulted in higher live births compared to CC with metformin (32, 39), letrozole resulted in higher pregnancy and ovulation than anastrozole and higher ovulation than LOD (49). Long-term letrozole (10 days) resulted in higher pregnancy than short-term letrozole (5 days) (Tables1, 2) (49). 

**Table 1:** Results of main medical interventions


Review	Population	Outcomes assessed	Comparison	Outcomes with significant results	
Letrozole					

Abu Hashim et al. (32), 2015	CC resistant PCOS	Live birth	CC+metformin vs. Letrozole	Live birth/woman	OR: 0.21, 95% CI: 0.05 to 0.87
		Pregnancy			
		Ovulation			
		Miscarriage			
		Multiple pregnancy			
		OHSS			
Franik et al. (39), 2014	PCOS, reproductive age	Live birth	Letrozole vs. CC (BMI >25 kg/m^2^)	Live birth/woman	OR: 1.67, 95% CI: 1.31 to 2.11
		Pregnancy	Letrozole vs. CC (with or without adjuncts followed by timed intercourse)	Live birth/woman	OR: 1.64, 95% CI: 1.32 to 2.04
		Miscarriage	Letrozole vs. CC (with or without adjuncts followed by IUI)	Pregnancy/woman	OR: 1.71, 95% CI: 1.30 to 2.25
		Multiple pregnancy	Letrozole vs. CC (overall with or without adjuncts followed by timed intercourse)	Pregnancy/woman	OR: 1.40, 95% CI: 1.18 to 1.65
		OHSS	Letrozole vs. CC+rFSH and rFSH only	Pregnancy/woman	OR: 1.66, 95% CI: 1.23 to 2.22
			**Letrozole+metformin vs. CC+metformin **	Live birth/woman	OR: 4.5, 95% CI: 1.09 to 18.50
He and Jiang (20), 2011	PCOS	Pregnancy	Letrozole vs. CC	Mature follicles/cycle	SMD: 1.41, 95% CI: 1.54 to 1.28
		Ovulation			
		Miscarriage			
		Multiple pregnancy			
		OHSS	Letrozole vs. CC	Ovulation/cycle	RR: 1.29, 95% CI: 1.12 to 1.49
		Mature follicles			
Misso et al. (49), 2012	PCOS	Live birth	Letrozole long-term (10 days) vs. Letrozole short-term (5 days)	Pregnancy/cycle	Higher in long-term (10 days)
		Pregnancy	Letrozole vs. Anastrozole	Ovulation/cycle	Higher in letrozole
		Ovulation	Letrozole vs. Anastrozole	Pregnancy/woman	Higher in letrozole
		Miscarriage	Letrozole vs. CC	Ovulation/woman	OR: 2.90, 95% CI: 1.72 to 4.88
		Multiple pregnancies	Letrozole vs. LOD	Ovulation/cycle	Higher in letrozole
		Adverse events			
		Cost effectiveness			
Roque et al. (27), 2015	PCOS (therapy naïve)	Live birth	Letrozole vs. CC	Live birth/woman	RR: 1.55, 95% CI: 1.26 to 1.90
		Clinical pregnancy			
		Ovulation			
		Miscarriage			
		**Multiple pregnancy**	Letrozole vs. CC	Pregnancy/woman	RR: 1.38, 95% CI: 1.05 to 1.83
**CC**					
Abu Hashim et al. (32), 2015	CC resistant PCOS	Live birth	CC+metformin vs. Gonadotrophins	Live birth/woman	OR: 0.33, 95% CI: 0.13 to 0.85
		Pregnancy	CC+metformin vs. Gonadotrophins	Ovulation/woman	OR: 0.25, 95% CI: 0.15 to 0.41
		Ovulation	CC+metformin vs. CC + NAC	Ovulation/woman	OR: 8.93, 95% CI: 4.61 to 17.32
		Miscarriage	CC+metformin vs. Gonadotrophins	Pregnancy/woman	OR: 0.45, 95% CI: 0.27 to 0.75
		Multiple pregnancy	CC+metformin vs. CC + NAC	Pregnancy/woman	OR: 5.28, 95% CI: 1.91 to 14.62
		OHSS			
Brown and Farquhar (46), 2017	WHO group 2 anovulation	Live birth	CC vs. Placebo	Pregnancy/woman	OR: 5.91, 95% CI: 1.77 to 19.68
		Pregnancy	CC vs. Gonadotrophins	Live birth/woman	OR: 0.64, 95% CI: 0.41 to 0.98
		Ovulation	CC vs. Gonadotrophins	Pregnancy/woman	OR: 0.61, 95% CI: 0.40 to 0.93
		Miscarriage	CC 5 day vs. CC 10 day	Live birth/woman	OR: 0.10, 95% CI: 0.02 to 0.45
		Multiple pregnancy	CC 5 day vs. CC 10 day	Pregnancy/woman	OR: 0.18, 95% CI: 0.06 to 0.55
		OHSS	CC+DEX vs. CC	Pregnancy/woman	OR: 6.2, 95% CI: 2.20 to 17.48
		Adverse effects	Early CC vs. late CC	Pregnancy/woman	OR: 2.81, 95% CI: 1.02 to 7.75
			CC+OCP vs. CC	Pregnancy/woman	OR: 27.18, 95% CI: 3.14 to 235.02
Ding et al. (36), 2016	PCOS	Pregnancy	Late CC vs. Early CC	Mature follicles/cycle	MD: 1.82, 95% CI: 0.86 to 2.78
		Ovulation			
		Miscarriage			
		Number of follicles			
Farquhar et al. (19), 2012	CC resistant PCOS	Live birth	LOD vs. CC+metformin	Live birth/woman	OR: 0.44, 95% CI: 0.24 to 0.82
		Pregnancy	LOD vs. CC+metformin	Costs	MD: 3711.3, 95% CI: 3585.17 to 3837.43
		Ovulation			
		Miscarriage			
		Multiple pregnancy			
		OHSS			
		Costs			
Gill et al. (33), 2014	CC resistant PCOS, reproductive age	Pregnancy	CC+metformin vs. CC	Ovulation/woman	Higher in CC+metformin
		Ovulation	CC+metformin vs. CC	Pregnancy/woman	Higher in CC+metformin
Palomba et al. (24), 2009	PCOS	Live birth	Metformin vs. CC+metformin	Live birth/woman	OR: 0.23, 95% CI: 0.13 to 0.40
		Pregnancy	Metformin vs. CC+metformin	Ovulation/woman	OR: 0.23, 95% CI: 0.15 to 0.34
		Ovulation	Metformin vs. CC+metformin	Pregnancy/woman	OR: 0.23, 95% CI: 0.14 to 0.37
		Miscarriage			
		Adverse events			
Siebert et al. (28), 2012	PCOS (therapy naïve)	Live birth	Metformin vs. CC	Live birth/woman	OR: 0.48, 95% CI: 0.31 to 0.73
		Pregnancy	Metformin vs. CC	Ovulation/woman	OR: 0.48, 95% CI: 0.41 to 0.57
		Ovulation	CC+metformin vs. CC	Ovulation/woman	OR: 1.6, 95% CI: 1.2 to 2.1
			CC+metformin vs. CC	Pregnancy/woman	OR: 1.3, 95% CI: 1.0 to 1.6
Tang et al. (41), 2012	PCOS	Live birth	Metformin vs. CC (BMI≥ 30)	Live birth/woman	OR: 0.3, 95% CI: 0.17 to 0.52
			Metformin vs. CC (BMI ≥ 30)	Ovulation/woman	OR: 0.43, 95% CI: 0.36 to 0.51
		Pregnancy	CC+metformin vs. CC (CC resistant PCOS)	Ovulation/woman	OR: 4.86, 95% CI: 2.43 to 9.74
		Ovulation	CC+metformin vs. CC (BMI<30)	Ovulation/woman	OR: 1.75, 95% CI: 1.27 to 2.39
		Miscarriage	CC+metformin vs. CC (BMI≥30)	Ovulation/woman	OR: 1.78, 95% CI: 1.51 to 2.1
		Multiple pregnancy	Metformin vs. CC (BMI ≥ 30)	Pregnancy/woman	OR: 0.34, 95% CI: 0.21 to 0.55
		Menstrual frequency	Metformin vs. CC (BMI <30)	Pregnancy/woman	OR: 1.94, 95% CI: 1.19 to 3.16
			CC+metformin vs. CC	Pregnancy/woman	OR: 1.51, 95% CI: 1.17 to 1.96
			CC+metformin vs. CC (BMI ≥30)	Pregnancy/woman	OR: 1.76, 95% CI: 1.26 to 2.47
			CC+metformin vs. CC	Side effects	OR: 3.31, 95% CI: 2.11 to 5.20
			CC+metformin vs. CC	Side effects (GIT)	OR: 3.4, 95% CI: 2.08 to 5.54
Thakker et al. (47), 2015	PCOS	Live birth	NAC vs. Placebo (CC resistant PCOS)	Live birth/woman	OR: 3.0, 95% CI: 1.05 to 8.6
		Ovulation	NAC vs. Placebo (CC resistant PCOS)	Ovulation/woman	OR: 8.4, 95% CI: 4.5 to 15.67
		Miscarriage, Multiple pregnancy OHSS	NAC vs. Placebo (CC resistant PCOS)	Pregnancy/woman	OR: 4.83, 95% CI: 2.30 to 10.13
		Menstrual regularity			
Xiao et al. (3), 2012	PCOS, <35 years	Pregnancy	Metformin vs. CC	Ovulation/woman	OR: 0.48, 95% CI: 0.26 to 0.87
		Ovulation	Metformin+CC vs. CC	Pregnancy/woman	OR: 1.56, 95% CI: 1.16 to 2.08
		Miscarriage			
**Insulin sensitizers**					
Tang et al. (41), 2012	PCOS	Live birth	Metformin vs. Placebo	Side effects (GIT)	OR: 4.27, 95% CI: 2.4 to 7.59
		Clinical pregnancy	Metformin vs. Placebo (BMI < 30)	Menstrual frequency	OR: 21.15, 95% CI: 1.01 to 445.0
		Ovulation	Metformin vs. Placebo (BMI ≥30)	Menstrual frequency	OR: 1.57, 95% CI: 1.03 to 2.41
		Miscarriage	Metformin vs. Placebo (BMI<30)	Pregnancy/woman	OR: 2.35, 95% CI: 1.44 to 3.82
		Multiple pregnancy	Metformin vs. Placebo	Menstrual frequency	OR: 1.72, 95% CI: 1.14 to 2.61
		Menstrual frequency	Metformin vs. Placebo	Ovulation/woman	OR: 1.81, 95% CI: 1.13 to 2.93
			Metformin vs. Placebo	Pregnancy/woman	OR: 2.31, 95% CI: 1.52 to 3.51
Feng et al. (52), 2015	PCOS, pregnant and took metformin to get conception	GDM, PE, Miscarriage, Premature delivery	Metformin during pregnancy vs. Placebo	Miscarriage	RR: 0.32, 95% CI: 0.19 to 0.56
			Metformin during pregnancy vs. Placebo	Preterm birth	RR: 0.4, 95% CI: 0.18 to 0.91
Tan et al. (58), 2016	PCOS and pregnant	GDM, PIH/PE, Miscarriage Preterm delivery	Metformin during pregnancy vs. Placebo	GDM	OR: 0.28, 95% CI: 0.10 to 0.75
			Metformin during pregnancy vs. Placebo	Miscarriage	OR: 0.20, 95% CI: 0.12 to 0.31
		Fetal abnormality, Fetal birth weight	Metformin during pregnancy vs. Placebo	Preterm birth	OR: 0.33, 95% CI: 0.18 to 0.60
			Metformin during pregnancy vs. Placebo (Non RCTs)	GDM	OR: 0.14, 95% CI: 0.09 to 0.24
			Metformin during pregnancy vs. Placebo (Non RCTs)	PIH/PE	OR: 0.28, 95% CI: 0.16 to 0.48
Zhuo et al. (54), 2014	PCOS and pregnant	GDM	Metformin during pregnancy vs. Placebo	GDM	OR: 0.19, 95% CI: 0.13 to 0.27
Zeng et al. (53), 2016	PCOS and pregnant	Live birth	Metformin during pregnancy vs. Placebo	GDM	OR: 0.02, 95% CI: 0.14 to 0.87
		Miscarriage	Metformin during pregnancy vs. Placebo	IUGR	OR: 0.17, 95% CI: 0.08 to 0.33
		Preterm delivery GDM	Metformin during pregnancy vs. Placebo	Live birth/woman	OR: 5.23, 95% CI: 3.12 to 8.75
		PIH/PE	Metformin during pregnancy vs. Placebo	Miscarriage	OR: 0.19, 95% CI: 0.12 to 0.28
		IUGR	Metformin during pregnancy vs. Placebo	PIH/PE	OR: 0.22, 95% CI: 0.13 to 0.38
		Fetal malformation	Metformin during pregnancy vs. Placebo	Preterm birth	OR: 0.37, 95% CI: 0.20 to 0.68
		Neonatal death Macrosomia			
Li et al. (22), 2011	PCOS	Pregnancy	Metformin vs. Thiazolidinediones (3 months duration)	Side effects	OR: 8.88, 95% CI: 3.54 to 22.27
		Ovulation			
		Menstrual regularity	Metformin vs. Thiazolidinediones (6 months duration)	Side effects	OR: 12.22, 95% CI: 3.53 to 42.31
Thakker et al.(47), 2015	PCOS	Live birth	NAC vs. Metformin	Ovulation/woman	OR: 0.13, 95% CI: 0.08 to 0.22
		Ovulation	NAC vs. Metformin	Pregnancy/woman	OR: 0.4, 95% CI: 0.23 to 0.71
		Miscarriage, Multiple pregnancy OHSS			
		Menstrual regularity			
Al Khalifah et al. (34), 2016	Adolescents with PCOS (11-19 year old)	Menstrual regulation	OCP vs. Metformin	Menstrual frequency	MD; 0.27, 95% CI: -0.33 to -0.21
Fang et al. (37), 2017	PCOS	Dominant follicles Menstrual regularity	Vitamin D + metformin vs. Metformin	Menstrual frequency	OR: 1.85, 95% CI: 1.01 to 3.39
Pundir et al. (38), 2017	PCOS	Live birth Clinical pregnancy	Inositol vs. Placebo	Ovulation/woman	RR: 2.3, 95% CI: 1.1 to 4.7
		Ovulation Miscarriage	Inositol vs. Placebo	Menstrual frequency	RR: 6.8, 95% CI: 2.8 to 16.6
		Menstrual regulation	Pioglitazone vs. Placebo	Menstrual frequency	OR: 8.88, 95% CI: 2.35 to 33.61
			Roziglitazone vs. Placebo	Menstrual frequency	OR: 5.59, 95% CI: 2.20 to 14.19


BMI; Body mass index, CC; Clomiphene citrate, DEX; Dexamethasone, GDM; Gestational diabetes mellitus, GIT; Gastrointestinal tract, IUGR; Intra-uterine growth restriction, IUI; Intra uterine insemination, LOD; Laparoscopic ovarian drilling, MD; Mean difference, NAC; N-acetyl cysteine, OCP; Oral contraceptive pills, OHSS; Ovarian hyper-stimulation syndrome, OR: Odds ratio, PCOS; Polycystic ovary syndrome, PIH/PE; Pregnancy induced hypertension/Preeclampsia, RCT; Randomized controlled trial, rFSH: Recombinant follicle stimulating hormone, RR; Risk ratio, SMD; Standardized mean difference, and WHO; World Health Organization.

**Table 2:** Results of their interventions


Gonadotrophins					

Bordewijk et al. (45), 2017	PCOS and anovulatory women	Live birth	FSH+metformin vs. FSH in PCOS resistant	Live birth/woman	OR: 2.31, 95% CI: 1.23 to 4.34
		Clinical pregnancy	FSH+metformin vs. FSH in PCOS resistant	Pregnancy/woman	OR: 2.46, 95% CI: 1.36 to 4.46
		Ovulation			
		Multiple pregnancy Miscarriage			
		OHSS			
		Adverse effects			
Farquhar et al. (19), 2012	CC resistant PCOS	Live birth	LOD vs. Gonadotrophins long-term	Costs	MD: -2235.0, 95% CI: -4433.16 to -36.84
		Pregnancy	LOD vs. Gonadotrophins short-term	Costs	MD: -1115.75, 95% CI: -1309.72 to -921.77
		Ovulation			
		Miscarriage			
		Multiple pregnancy			
		OHSS	LOD vs. Gonadotrophins	Multiple pregnancy	OR: 0.13, 95% CI: 0.03 to 0.52
		Costs			
Moazami et al. (23), 2014	CC-resistant PCOS	Live birth Pregnancy Miscarriage	LOD vs. Gonadotropins	Live birth/woman	OR: 0.446, 95% CI: 0.269 to 0.74
		Multiple pregnancies	LOD vs. Gonadotropins	Multiple pregnancy	OR: 0.127, 95% CI: 0.028 to 0.579
		Multiple pregnancy OHSS	Gonadotrophins+metformin vs. Gonadotrophins in OI	Pregnancy/woman	OR: 2.25, 95% CI: 1.50 to 3.38
			Gonadotrophins+metformin vs. Gonadotrophins in OI	Cancellation/cycle	OR: 0.41, 95% CI: 0.24 to 0.72
			Gonadotrophins+metformin vs. Gonadotrophins in OI	Gonadotrophins units	MD: 306.62, 95% CI: -500.02 to -113.22
			Gonadotrophins+metformin vs. Gonadotrophins in OI	Stimulation length	MD: -3.28, 95% CI: -6.23 to -0.32
Palomba et al. (40), 2014	PCOS	Live birth Pregnancy Miscarriage	Gonadotrophins+metformin vs. Gonadotrophins in OI	Live birth/woman
		Multiple pregnancy OHSS	Gonadotrophins+metformin vs. Gonadotrophins in OI	Pregnancy/woman
			Gonadotrophins+metformin vs. Gonadotrophins in OI	Cancellation/cycle
			Gonadotrophins+metformin vs. Gonadotrophins in OI	Gonadotrophins units
			Gonadotrophins+metformin vs. Gonadotrophins in OI	Stimulation length
Weiss et al. (29), 2015	CC-resistant ± failure PCOS Women treated with prior metformin use +/- CCWomen with prior electro cautery of ovaries.	Live birth	rFSH vs. All urinary gonadotrophins	Gonadotrophins units	MD: -105.44, 95% CI: -154.21, -56.68
			rFSH vs. HMG	Gonadotrophins units	MD: -283.94, 95% CI: -449.10 to -118.78
		Clinical pregnancy Miscarriage	rFSH vs. uFSH	Gonadotrophins units	MD: -88.4, 95% CI: -139.44 to -37.36
			rFSH vs. All urinary gonadotrophins	Stimulation length	MD: -0.66, 95% CI: -1.04 to -0.28
		Multiple pregnancy OHSS	rFSH vs. HMG	Stimulation length	MD: -2.28, 95% CI: -3.49 to -1.07
			rFSH vs. uFSH	Stimulation length	MD: -0.49, 95% CI: -0.88 to -0.09
**Laparoscopic ovarian drilling (LOD)**					
Farquhar et al. (19), 2012	CC resistant PCOS	Live birth	LOD vs. Metformin	Pregnancy/woman	OR: 2.47, 95% CI: 1.05 to 5.81
		Pregnancy	LOD vs. Other medical treatments	Multiple pregnancy	OR: 0.21, 95% CI: 0.08 to 0.58
		Ovulation			
		Miscarriage			
		Multiple pregnancy			
		OHSS			
		Costs			
Baghdadi et a. (56), 2012	CC resistant PCOS	Pregnancy	Lean vs. Obese PCOS	Ovulation/cycle	RR: 1.90, 95% CI: 1.46 to 2.48
			Lean vs. Obese PCOS	Ovulation/woman	RR: 1.43, 95% CI: 1.22 to 1.66
		Ovulation	Lean vs. Obese PCOS	Pregnancy/cycle	RR: 4.14, 95% CI: 2.08 to 8.23
			Lean vs. Obese PCOS	Pregnancy/woman	RR: 1.73, 95% CI: 1.39 to 2.17
**IUI/IVF/ICSI related interventions**					
Luo et al. (57), 2014	PCOS undergoing COS/IUI	Live birth	GnRH antagonist +IUI vs. Control IUI	LH	MD: 4.6, 95% CI: 0.9 to 8.31
		Clinical pregnancy	GnRH antagonist +IUI vs. Control IUI	Premature lutenization rate	OR: 4.36, 95% CI: 2.15 to 8.84
		Miscarriage	GnRH antagonist +IUI vs. Control IUI	Progesterone	MD: 0.31, 95% CI: 0.24 to 0.37
Kollman et al. (18), 2016	PCOS		Inositol vs. Placebo IVF	Pregnancy/woman	RR: 1.41, 95% CI: 1.05 to 1.89
		Live birth/ ongoing pregnancy	Myo-inositol vs. D-chiro-inositol	Pregnancy/woman	RR: 2.86, 95% CI: 1.14 to 7.16
		Clinical pregnancy Miscarriage	Antagonist vs. Agonist	OHSS	RR: 0.63, 95% CI: 0.49 to 0.80
		OHSS	Mannitol vs. Placebo	OHSS	RR: 0.54, 95% CI: 0.39 to 0.77
Palomba et al. (59), 2013	PCOS undergoing IVF cycles	Live birth Pregnancy Miscarriage	Gonadotrophins+metformin vs. Gonadotrophins (Metformin stopping time until 12 weeks of gestation)	Live birth/woman	OR: 75.6, 95% CI: 8.03 to 711.5
			Gonadotrophins+metformin vs. Gonadotrophins	Miscarriage	OR: 0.50, 95% CI: 0.30 to 0.83
			Gonadotrophins+metformin vs. Gonadotrophins (Metformin stopping time until 12 weeks of gestation)	Miscarriage	OR: 0.08, 95% CI: 0.02 to 0.39
			Gonadotrophins+metformin vs. Gonadotrophins (Pretreatment length effect for long-term >3 weeks)	Miscarriage	OR: 0.41, 95% CI: 0.21 to 0.78
			Gonadotrophins+metformin vs. Gonadotrophins (Pretreatment length effect for short-term ≤ 3 weeks)	OHSS	OR: 0.20, 95% CI: 0.07 to 0.54
			Gonadotrophins+metformin vs. Gonadotrophins (Metformin stopping time until oocyte retrieval, ET and HCG injection)	OHSS	OR: 0.22, 95% CI: 0.11 to 0.42
			Gonadotrophins+metformin vs. Gonadotrophins (no pretreatment period)	OHSS	OR: 0.14, 95% CI: 0.05 to 0.38
			Gonadotrophins+metformin vs. Gonadotrophins (higher dose >1000 mg daily)	OHSS	OR: 0.40, 95% CI: 0.20 to 0.80
			Gonadotrophins+metformin vs. Gonadotrophins (lower dose <=1000 mg/daily)	OHSS	OR: 0.15, 95% CI: 0.06 to 0.38
		OHSS	Gonadotrophins+metformin vs. Gonadotrophins	OHSS	OR: 0.27, 95% CI: 0.16 to 0.46
			Gonadotrophins+metformin vs. Gonadotrophins	Oocyte number retrieved	WMD: -1.11, 95% CI: -1.86 to -0.36
			Gonadotrophins+metformin vs. Gonadotrophins (higher dose >1000 mg daily)	Oocyte number retrieved	WMD: -1.16, 95% CI: -1.96 to -0.37
			Gonadotrophins+metformin vs. Gonadotrophins (Pretreatment length effect for long-term >3 weeks)	Oocyte number retrieved	WMD: -1.45, 95% CI: -2.37 to -0.53
			Gonadotrophins+metformin vs. Gonadotrophins (Metformin stopping time until pregnancy test)	Oocyte number retrieved	WMD: -1.32, 95% CI: -2.40 to -0.23
			Gonadotrophins+metformin vs. Gonadotrophins (Pretreatment length effect for long-term >3 weeks)	Implantation/embryo	OR: 0.28, 95% CI: 0.12 to 0.62
			Gonadotrophins+metformin vs. Gonadotrophins (higher dose > 1000 mg daily)	Implantation/embryo	OR: 1.42, 95% CI: 1.24 to 2.75
			Gonadotrophins+metformin vs. Gonadotrophins (Metformin stopping time until pregnancy test)	Stimulation length	WMD: 0.85, 95% CI: 0.02 to 1.68
			Gonadotrophins+metformin vs. Gonadotrophins (lower dose <=1000 mg/daily)	Gonadotrophins units	WMD: -326.84, 95% CI: -505.99 to -147.69
Huang et al. (21), 2015	PCOS undergoing IVF/ICSI in non-donor cycles	Live birth Clinical pregnancy	Metformin vs. Placebo	OHSS	RR: 0.44; 95%CI 0.26 to 0.77
		Miscarriage Multiple pregnancy			
		OHSS			
Tso et al. (42), 2014	PCOS and of reproductive age undergoing IVF or ICSI	Live birth	Metformin vs. Placebo	Pregnancy/woman	OR: 1.52, 95% CI: 1.07 to 2.15
		Clinical pregnancy	Metformin vs. Placebo	Side effects	OR: 4.49, 95% CI: 1.88 to 10.72
		Miscarriage OHSS	Metformin vs. Placebo	OHSS	OR: 0.29, 95% CI: 0.18 to 0.49
		Side effects	Metformin vs. Placebo (long protocol GnRH agonist)	OHSS	OR: 0.29, 95% CI: 0.16 to 0.51
Pundir et al. (26), 2012	PCOS undergoing IVF with or without ICSI	Live birth	GnRH antagonist vs. Agonist	Gonadotrophins units	WMD: -0.28, 95% CI: -0.43 to -0.13)
		Clinical pregnancy	GnRH antagonist vs. Agonist	Moderate and severe OHSS	RR: 0.59, 95% CI: 0.45 to 0.76
		Ongoing pregnancy	GnRH antagonist vs. Agonist	OHSS (moderate & severe)	RR: 0.60, 95% CI: 0.48 to 0.76
		Miscarriage	GnRH antagonist vs. Agonist	Stimulation length	WMD: -0.74, 95% CI: -1.12 to -0.36
		OHSS			
Siristatidis et al. (50), 2015	PCOS, PCO and control undergoing IVM	Live birth	IVM in (PCOS vs. Control)	Cancellation/cycle	OR: 0.15, 95% CI: 0.05 to 0.44
		Clinical pregnancy	IVM in (PCOS vs. Non PCOS)	Cancellation/cycle	OR: 0.18, 95% CI: 0.06 to 0.47
		Miscarriage	IVM in (PCOS vs. PCO)	Cancellation/cycle	OR: 0.25, 95% CI: 0.07 to 0.92
		Oocyte maturation	IVM in (PCOS vs. Non PCOS)	Implantation/embryo	OR: 1.73, 95% CI: 1.06 to 2.81
			IVM in (PCOS vs. Control)	Maturation/oocyte	OR: 0.74, 95% CI: 0.59 to 0.93
			IVM in (PCOS vs. Control)	Pregnancy/cycle	OR: 3.09, 95% CI: 1.46 to 6.53
			IVM in (PCOS vs. Non-PCOS)	Pregnancy/cycle	OR: 2.23, 95% CI: 1.45 to 3.43
			IVM in (PCOS vs. Control)	Pregnancy/woman	OR: 3.29, 95% CI: 1.42 to 7.62
			IVM in (PCOS vs. Non PCOS)	Pregnancy/woman	OR: 2.37, 95% CI: 1.53 to 3.68
Xiao et al. (31), 2013	PCOS	Clinical pregnancy	GnRH antagonist vs. GnRH agonist	Moderate-severe OHSS	OR: 0.36, 95% CI: 0.25 to 0.52


CC; Clomiphene citrate, COS; Controlled ovarian stimulation, ET; Embryo transfer, FSH: Follicle stimulating hormone, GnRH; Gonadotrophins releasing hormone, HCG; Human chorionic gonadotrophin, HMG; Human menopausal gonadotrophin, ICSI; Intra cytoplasmic sperm injection, IUI; Intra uterine insemination, IVF; In vitro fertilization, IVM; In vitro maturation, LH; Luteinizing hormone, LOD; Laparoscopic ovarian drilling, MD; Mean difference, OHSS; Ovarian hyper-stimulation syndrome, OI; Ovulation induction, OR; Odds ratio, PCOS; Polycystic ovary syndrome, rFSH; Recombinant follicle stimulating hormone, RR; Risk ratio, uFSH; Urinary follicle stimulating hormone, and WMD; Weighted mean difference

### Clomiphene citrate

Seventeen reviews, [seven high quality (19, 39, 41, 46- 49), six moderate quality (16, 20, 24, 27, 32, 38) and four low quality (28, 30, 33, 36)] assessed interventions that contained CC, comprising a total of 203 trials with 26 731 participants. One review assessed CC versus LOD (19). One review assessed early follicular versus late luteal CC administration (36). The remaining 14 reviews assessed CC ± other OI drugs such as metformin, inositol, N-acetyl cysteine (NAC) and others versus other OI drugs, including CC. The populations studied were women with PCOS who were treatment-naïve (27), CC resistant (19, 32, 33) and women with PCOS who were treatment-naïve ± CC resistant PCOS or unknown treatment status. 

The meta-analyses reported in overall women with PCOS that CC compared to placebo had statistically higher pregnancy and ovulation (46). Early follicular CC had higher pregnancy than late luteal CC (46) but with less mature follicles (36). Higher live birth, pregnancy, and ovulation resulted after CC compared to metformin mainly in women with BMI ≥30 kg/m^2^ (28, 30, 41) while metformin resulted in higher pregnancy than CC in women with BMI <30 kg/m^2^ (41). CC plus metformin was of more benefit than CC or metformin alone with regards to live birth (24), pregnancy and ovulation, but had higher gastrointestinal side effects (24, 28, 30, 33, 41). Higher live birth and pregnancy resulted after gonadotrophins compared to CC and 10 days of CC compared to 5 days of CC, respectively (46).

In women with CC resistant PCOS, gonadotrophins resulted in statistically higher live birth, pregnancy and ovulation than CC plus metformin (32, 46) which, in turn, resulted in higher live birth than LOD (19). In the same population of women, the addition of dexamethasone, NAC or contraceptive pills to CC resulted in higher live births, pregnancy and ovulation than CC alone (46, 47). Furthermore, the addition of metformin to CC resulted in more favourable outcomes compared with the addition of NAC with regards to pregnancy and ovulation. However, the cost of treatment was greater for gonadotrophins followed by LOD then CC plus metformin (19).

### Gonadotrophins

Ten reviews [six high quality (19, 29, 39, 45, 46, 49) and four moderate quality (23, 32, 40, 59)] assessed interventions containing gonadotrophins, which comprised 146 trials with 18 379 participants. Two reviews assessed gonadotrophins versus LOD (19, 23). Three reviews assessed the effectiveness of adding metformin to gonadotrophins during OI (40, 45) and IVF (59). Two reviews assessed gonadotrophins versus anti-oestrogens ± adjunctive drugs (32, 46). Two reviews assessed gonadotrophins versus aromatase inhibitors (39, 49). One review assessed the effectiveness of different types of gonadotrophins (29). The populations studied were women with CC resistant PCOS (19, 23, 29, 32) and women who were treatment-naïve ± CC resistant PCOS women or unknown treatment status.

The meta-analyses reported that in women with CC resistant PCOS, gonadotrophins resulted in statistically higher live births, multiple pregnancies, and costs of short- and long-term treatment in comparison to LOD (19, 23) and higher live births, pregnancy and ovulation in comparison to CC ± metformin (32, 46), but lower pregnancy in comparison to letrozole (39). Adding metformin to gonadotrophins, compared to gonadotrophins alone, resulted in higher live birth and pregnancy in OI (40, 45) and higher live birth, implantation rate, lower miscarriage, ovarian hyperstimulation syndrome (OHSS) and number of oocyte retrieved in IVF (59). Recombinant follicle stimulating hormone (FT.) resulted in lower dose and stimulation duration than other urinary gonadotrophins in OI (29).

### Insulin sensitizers

Thirty reviews (12 reviews of high quality (18, 19, 34, 39, 41, 42, 44-49), 13 reviews of moderate quality (16, 21, 22, 24, 25, 32, 35, 37, 38, 40, 53, 54, 59) and five reviews of low quality (28, 30, 33, 52, 58) assessed interventions that contained insulin sensitizers comprising 398 trials with 45 031 participants. Four reviews assessed metformin versus placebo (18, 21, 41, 42). Four reviews assessed metformin during pregnancy (52-54, 58). One review assessed the effect of pre-gestational metformin on risk of miscarriage (25). One review assessed roziglitazone, plioglitazone, and D-chiro-inositol versus placebo (41). One review assessed metformin versus thiazolidinediones (22). One review assessed LOD versus metformin (19). One review assessed NAC versus placebo or metformin (47). One review assessed oral contraceptive pills versus metformin (34). One review assessed the benefit of adding vitamin D to metformin (37).Three reviews had CC resistant PCOS women as participants (19, 32, 33) while the others did not clarify the treatment status.

The meta-analyses reported that, overall in women with PCOS, metformin resulted in higher live births, pregnancy, and gastrointestinal side effects with lower OHSS than placebo when used in addition to IVF (18, 21, 42) and higher pregnancy, ovulation, side effects and menstrual frequency in OI (41). Metformin had higher gastrointestinal side effects than thiazolidinediones (22). In women with CC resistant PCOS, NAC resulted in higher live births, pregnancy and ovulation than placebo, but lower pregnancy and ovulation than metformin (47). Oral contraceptive pills were better than metformin in improving menstrual frequency (34). Adding vitamin D to metformin improved menstrual frequency than metformin alone (37). Inositol resulted in higher pregnancy than placebo with more benefit of myoinositol over D-chiro inositol in IVF (18), while inositol resulted in higher ovulation than placebo in OI. Roziglitaone, pioglitazone and inositol improved menstrual frequency in OI (38). In women with PCOS who became pregnant, metformin intake during pregnancy resulted in higher live birth and lower miscarriage, preterm labour, gestational hypertension, preeclampsia, gestational diabetes and intrauterine growth retardation (52-54, 58).

### Laparoscopic ovarian drilling

Six reviews [four high quality (19, 32, 39, 49) and two moderate quality (23, 56)] assessed ovarian ablation therapy and LOD as an intervention in PCOS comprising 97 trials with 13 617 participants. Three reviews had participants as CC resistant PCOS (19, 23, 56).

The meta-analyses reported that LOD resulted in lower live births than CC plus metformin and gonadotrophins, respectively (19, 23), higher pregnancy than metformin alone (19), lower ovulation than letrozole (49), higher costs than CC plus metformin but lower than gonadotrophins (19) and lower multiple pregnancy rate than other medical treatments (19). Pregnancy and ovulation were higher in lean women (BMI <25 kg/m^2^) with CC resistant PCOS than in overweight and obese women (BMI ≥25 kg/m^2^) undergoing LOD (56). 

### Intrauterine insemination, *in vitro* fertilization, intracytoplasmic sperm injection related interventions

Nine reviews [three high quality (17, 18, 42)] and six moderate quality (21, 26, 31, 50, 57, 59) assessed different interventions in women with PCOS undergoing assisted reproductive techniques [intrauterine insemination (IUI), IVF/ICSI] comprising 126 trials with 12 298 participants in eight reviews and 333 cycles in the ninth review which did not report on the number of participants (57). Three reviews assessed gonadotrophin releasing hormone (GnRH) antagonist as an adjuvant intervention in controlled ovarian stimulation plus IUI (57) and in comparison with GnRH agonist during IVF/ICSI (26, 31). Three reviews assessed the effect of metformin during IVF/ICSI (21, 42, 59). Two reviews assessed the use of IVM (17, 50).

The meta-analyses reported statistically significant results for lower progesterone, luteinizing hormone (LH) and premature luteinisation rate during IUI after GnRH antagonist (57) and lesser dose, duration of gonadotrophins and OHSS rate after GnRH antagonist during lVF/ ICSI . Metformin compared to placebo in IVF resulted in higher live births (18, 59), pregnancy (18, 42), lower miscarriage (59), lower OHSS (18, 21, 42, 59), and lower oestradiol (E2), gonadotrophin dose and higher implantation rate (59); however, disadvantages included more, yet mild, gastrointestinal side effects (42). Compared to placebo, inositol resulted in higher pregnancy with better results after myoinositol than D-Chiro inositol, while mannitol resulted in lower OHSS (18). IVM used in women with PCOS had higher pregnancy, lower cancelled cycles, higher implantation but lower mature oocytes than IVM in non-PCOS patients (50).

### Other interventions

A low quality review reported that bariatric surgery improved menstrual frequency in women with PCOS in six trials and 264 participants (51). A high quality review reported that statins did not improve menstrual frequency or ovulation in women with PCOS not trying to conceive in four trials and 244 participants (43). A high quality review (44) assessed the use of antidepressants in women with PCOS, and identified no studies reporting on any of the primary reproductive outcomes with the exception of one RCT that reported on endocrine and metabolic outcomes between fluoxetine with sibutramine found no significant difference between both drugs (61). A moderate quality review assessed orlistat versus other anti-obesity drugs and found no difference in reproductive outcomes (55).

## Discussion

We reported the first overview of systematic reviews on treatment for reproductive outcomes in women with PCOS. This review follows a process of systematic reviews proposed by the Cochrane collaboration that summarizes evidence from more than one systematic review of different interventions for the same condition (62, 63). This type of review can be utilized as a rich source of data synthesis for developing and updating guidelines, and for health care policy makers. Our overview included 53 systematic reviews (9 older versions and 44 currently updated articles), 498 studies, and 56 057 participants. The quality of most included reviews was moderate to high, although the quality of included studies was variable.

Our results align with most current guidelines on PCOS. According to many guidelines, treatment of anovulation in PCOS should start with lifestyle modification before commencing pharmacological agents, especially in obese women with BMI >30 kg/m^2^ (1, 3, 8, 10, 11), The firstline pharmacological agent is usually CC (2, 3, 11, 64, 65) and some guidelines propose letrozole as an alternative (1, 8, 10). Our results suggest that, overall, in women with PCOS (with or without CC resistance), letrozole resulted in higher live birth and clinical pregnancy rates than other OI drugs, especially CC. This is consistent with many reviews and RCTs (9, 20, 27, 32, 39, 49, 66-68), despite the fact that letrozole is an off-label drug in OI. Nevertheless, the issue of safety in pregnancy for both CC and letrozole has not been completely resolved. Most large retrospective studies found no evidence of any difference between these drugs (69). Metformin is recommended in many guidelines as an adjunctive treatment with CC in women with glucose intolerance and in obese women (1-3, 8, 10), while the National Institute for Health and Clinical Excellence Guidance (NICE) recommended metformin alone or with CC as a first-line treatment (11). Our results suggest that, overall, in women with PCOS, CC plus metformin also resulted in in better reproductive outcomes than CC or metformin alone. The Australian National Health and Medical Research Council (NHMRC) evidence-based guidelines suggested that it is acceptable to use gonadotrophins as a first-line treatment (8). Our results suggest that the use of gonadotrophins resulted in higher live birth and clinical pregnancy rates than CC, overall, in women with PCOS.

CC is usually used for six months, which is recommended by many guidelines (1, 8, 11). After that, women are considered to be CC resistant, which necessitates a second-line treatment. Most fertility guidelines recommend low dose gonadotrophins or LOD as a second-line treatment (1-3, 8, 10, 11). CC plus metformin was also recommended by some guidelines, if not already used as a first-line treatment (8, 11). Gonadotrophins have the disadvantage of cost and increased rates of multiple pregnancies, while LOD has a risk with anaesthesia, decreased ovarian reserve, and the need to use adjuvant drugs for OI after surgery (3). Our results suggest that, in women with CC resistant PCOS, gonadotrophins resulted in better reproductive outcomes than many OI drugs with the disadvantages of increased multiple pregnancies and increased cost (19, 23, 32, 46). We found that women who used gonadotrophins had higher live birth than those who were prescribed CC plus metformin or LOD respectively, and higher clinical pregnancy and ovulation rates than CC plus metformin. CC plus metformin resulted in higher live birth rate and lower cost than LOD. Gonadotrophins are more expensive than LOD. LOD has the advantage of lower rates of multiple pregnancies compared to other interventions, such as gonadotrophins, in CC resistant PCOS (19). LOD in lean women seem to have better reproductive outcomes than in overweight and obese women.

Current recommendations state that IVF should be used in case of CC failure, which is defined by failure of conception after 6-9 months (1, 11). Our results support the current evidence for use of GnRH antagonists and addition of metformin to GnRH agonist to decrease OHSS (1). There is lack of data on use of IVM in PCOS (1), which is reported by one of included reviews (17). Another review by the same author reported higher pregnancy and implantation rates with lower cancellation rate in women with PCOS undergoing IVM compared to IVM in nonPCOS women (50).

Despite the large number of reviews and RCTs that have been conducted assessing different treatments for management of reproductive outcomes in women with PCOS, there are still a considerable number of research gaps. Recently, the international evidence-based guideline for the assessment and management of PCOS has issued new recommendations for the diagnosis and management of PCOS(70). These guidelines state that letrozole should be considered first-line pharmacological treatment for OI in women with PCOS with anovulatory infertility and no other infertility factors to improve ovulation, pregnancy and live birth rates. This is consistent with our results in this overview. They also stated that inositol (in any form) should currently be considered an experimental therapy in PCOS, with emerging evidence on efficacy highlighting the need for further research (70). Furthermore, research on the possible reasons for CC resistance and failure utilizing unified definitions is needed. This is particularly relevant given that some recent reviews revealed that the antiestrogenic effect of CC, specifically on endometrial tissue, is not enough rationale for resistance and failure (66). Furthermore, a recent crossover RCT found that there is no difference in clinical pregnancy and live birth rates between CC and letrozole when used as a second line treatment in women who failed to ovulate or conceive with CC or letrozole as first line of treatment (9). It is also important to note that a thorough study of the cost effectiveness of any of these treatments has not been performed, particularly in low income countries. Further investigation of metformin with regards to its cost effectiveness, safety, and effectiveness in non-obese women is also needed (1, 8). There is also a lack of data relating to the comparison between the use of LOD and medical treatment as a first line treatment, and the minimum efficient dose of LOD to induce ovulation without affecting ovarian reserve (1, 3, 11).

Limitations include our search strategy with reviews published from 2009 onwards, coinciding with the PRISMA statement publication for conducting systematic reviews. While this would miss earlier reviews, later included reviews would be likely to be of higher quality and aligned with the PRISMA statement. We applied language restrictions including only articles in English, which might lead to bias in exclusion of other languages. We found insufficient data on the quality of included studies in each review. We did not perform a quality assessment of each of the individual trials within each systematic review and relied instead on the judgement of the authors which varied from cursory to comprehensive; although we note that performing a quality assessment of 498 total studies would have been an extensive task. We note that the actual effect of different treatments in each treatment status and PCOS phenotypes is still unclear. We also note wide variability in the definition of outcomes across reviews and included studies. For instance, although pregnancy was reported as clinical pregnancy in most included reviews, ongoing pregnancy was reported in some reviews (26, 45) and pregnancy was not predefined in others (22, 24, 36, 40, 51, 56). The definition of clinical pregnancy varied across the included studies within each review.

## Conclusion

We report here a significant contribution to the literature in the overview and synthesis of systematic reviews that assessed medical and surgical treatments for reproductive outcomes in women with PCOS. In agreement with most recent international guidelines on management of PCOS, letrozole was superior to other OI agents as a first-line pharmacological treatment with gonadotrophins a second-line pharmacological treatment for anovulatory women with PCOS.

## Supplementary PDF


