# Supplementary material for: Medical and Surgical Treatment of Reproductive Outcomes in Polycystic Ovary Syndrome: An Overview of Systematic Reviews
Source: Int J Fertil Steril. 2019 Nov 11;13(4):257–70. doi: 10.22074/ijfs.2020.5608 (PMC6875858; doi:10.22074/ijfs.2020.5608)
Supplement: Supplementary file 1 [file Int-J-Fertil-Steril-13-257-s01.pdf]

Int J Fertil Steril
International Journal of Fertility & Sterility
ISSN:2008-076X eISSN:2008-0778

Supplementary Information for

Medical and Surgical Treatment of Reproductive Outcomes in Polycystic Ovary Syndrome: An Overview of Systematic Reviews

Moustafa A. Gadalla, M.Sc.[1, 2*], Robert J. Norman, M.D., FRANZCOG.[2], Chau T Tay, Ph.D.[3, 4], Danielle S. Hiam, Ph.D.[5], Angela Melder, Ph.D.[3], Jyotsna Pundir, M.D., Ph.D.[6], Shakila Thangaratinam, Ph.D.[7], Helena J Teede, Ph.D.[3, 4], Ben W. J. Mol, M.D., Ph.D.[2, 8], Lisa J. Moran, Ph.D.[2 ,3]

1. Women's Health Hospital, Department of Obstetrics and Gynaecology, Assiut University, Assiut, Egypt
2. Robinson Research Institute, Discipline of Obstetrics and Gynaecology, University of Adelaide, Adelaide, Australia
3. Monash Centre for Health Research and Implementation, School of Public Health and Preventive Medicine, Victoria, Melbourne, Australia
4. Monash Diabetes and Endocrinology Units, Monash Health, Victoria, Melbourne, Australia
5. Institute of Sport, Exercise and Active Living, Victoria University, Melbourne, Australia
6. Centre of Reproductive Medicine, St Bartholomew's Hospital, London, United Kingdom
7. Women's Health Research Unit, Barts and the London School of Medicine and Dentistry, Queen Mary University London, London, United Kingdom
8. Department of Obstetrics and Gynaecology, Monash University, Clayton, Victoria, Melbourne, Australia

Appendix 1: Search strategy

MEDLINE

1. exp Polycystic Ovary Syndrome/
2. Polycystic Ovar$.tw
3. pco.tw or pcos.tw
4. (sclerocystic adj3 ovar$).tw
5. stein leventhal.tw
6. or/1–5
7. Meta-Analysis as Topic/
8. meta analy$.tw
9. metaanaly$.tw
10. Meta-Analysis/
11. (systematic adj (review$1 or overview$1)).tw.
12. exp Review Literature as Topic/
13. or/7-12
14. cochrane.ab.
15. embase.ab.
16. (psychlit or psyclit).ab.
17. (psychinfo or psycinfo).ab.
18. (cinahl or cinhal).ab.
19. science citation index.ab.
20. bids.ab.
21. cancerlit.ab.
22. or/14-21
23. reference list$.ab.
24. bibliograph$.ab.
25. hand-search$.ab.
26. relevant journals.ab.
27. manual search$.ab.

*Corresponding Address: Department of Obstetrics and Gynecology, "Filippo Del Ponte" Hospital, University of Insubria, Piazza Biroldi 1, 21100, Varese, Italy
Email: antoniosimone.lagana@uninsubria.it

28. or/23-27
29. selection criteria.ab.
30. data extraction.ab.
31. 29 or 30
32. Review/
33. 31 and 32
34. Comment/
35. Letter/
36. Editorial/
37. animal/
38. human/
39. 37 not (37 and 38)
40. or/34-36,39
41. 13 or 22 or 28 or 33
42. 6 and 41
43. 42 not 40

Embase

1. exp Polycystic Ovary Syndrome/
2. Polycystic Ovar$.tw
3. pco.tw or pcos.tw
4. (sclerocystic adj3 ovar$).tw
5. stein leventhal.tw
6. or/1–5
7. exp Meta Analysis/
8. (meta adj analy$).tw
9. metaanalys$.tw.
10. (systematic adj (review$1 or overview$1)).tw.
11. or/7-10
12. cancerlit.ab.
13. cochrane.ab.
14. embase.ab.
15. (psychlit or psyclit).ab.
16. (psychinfo or psycinfo).ab.
17. (cinahl or cinhal).ab.
18. science citation index.ab.
19. bids.ab.
20. or/12-19
21. reference lists.ab.
22. bibliograph$.ab.
23. hand-search$.ab.
24. manual search$.ab.
25. relevant journals.ab.
26. or/21-25
27. data extraction.ab.
28. selection criteria.ab.
29. 27 or 28
30. review.pt.
31. 29 and 30
32. letter.pt.
33. editorial.pt.
34. animal/
35. human/
36. 34 not (34 and 35)
37. or/32-33,36
38. 11 or 20 or 26 or 31
39. 6 and 38
40. 39 not 37

Cinahl

1. MH “Polycystic Ovary Syndrome+”
2. TX Polycystic Ovar*
3. TX pco
4. TX pcos
5. TX stein leventhal
6. TX (sclerocystic n3 ovar*)
7. or/1–6
8. MH “Meta analysis+”
9. TX Meta analys*
10. TX Metaanaly*
11. MH “Literature review+”
12. TX (systematic adj (review or overview))
13. Or/8-12
14. PT Commentary
15. PT Letter
16. PT Editorial
17. MH “Animals+”
18. Or/14-17
19. 7 and 13
20. 19 not 18

PROSPERO

1. “PCOS”
2. “polycystic ovary syndrome”
3. 1 OR 2
4. TI 3

Appendix 2: Excluded studies

N.	ID	Author	Title	Excluded	Category	Notes
1.	1701	Abu Hashim (1), 2016	Twenty years of ovulation induction with metformin for PCOS; what is the best available evidence?	Yes	Fertility treatment	Didn't include quality assessment
2.	1704	Al Khalifah et al. (2), 2015	The effectiveness and safety of treatments used for polycystic ovarian syndrome management in adolescents: a systematic review and network meta-analysis protocol	Yes		Protocol only
3.		Al-Khalifah et al. (3), 2016	The effectiveness and safety of treatments used for polycystic ovarian syndrome management in adolescents: a systematic review and network meta-analysis.	Yes		Conference abstract only , no related publication found<br>No available online reference
4.	2179	Andrade (4), 2016	Major malformation risk, pregnancy outcomes, and neurodevelopmental outcomes associated with metformin use during pregnancy.	Yes		Not an original systematic review or meta-analysis
5.	1714	Atiomo et al. (5), 2009	Proteomic biomarkers for the diagnosis and risk stratification of polycystic ovary syndrome: a systematic review	Yes	Assessment	Didn't include quality assessment
6.	1722	Bagos (6), 2009	Plasminogen activator inhibitor-1 4G/5G and 5,10-methylene-tetrahydrofolate reductase C677T polymorphisms in polycystic ovary syndrome	Yes		Didn't include quality assessment or number of articles extracted on search
7.	1723	Bao et al. (7), 2016	Association of DENND1A Gene Polymorphisms with Polycystic Ovary Syndrome: A Meta-Analysis	Yes		Didn't include quality assessment
8.	1725	Baranova et al. (8), 2011	Systematic review: association of polycystic ovary syndrome with metabolic syndrome and non-alcoholic fatty liver disease	Yes	Assessment	Didn't include number of articles extracted on search or quality assessment
9.	1726	Barba et al. (9), 2009	The effects of metformin on endogenous androgens and SHBG in women: a systematic review and meta-analysis	Yes	Non-fertility medical treatment	Not primarily focused on PCOS
10.	1444	Bayram et al. (10) 2010	Pulsatile gonadotrophin releasing hormone for ovulation induction in subfertility associated with polycystic ovary syndrome	Yes	Fertility treatment	Although Cochrane assessed content as up to date in 2010, search was conducted in 2003 so deemed this as meeting prior to 2009 exclusion criteria
11.	1744	Birch Petersen et al. (11), 2016	Mono-ovulation in women with polycystic ovary syndrome: a clinical review on ovulation induction	Yes	Fertility treatment	Didn't include search terms or number of articles extracted on search
12.	1428	Bouza-Alvarez et al. (12), 2011	Safety and efficacy of metformin in improving clinical, hormonal and metabolic features of polycystic ovary syndrome. Systematic review and meta-analysis	Yes		? HTA, bulk of article in Spanish
13.	1750	Bronstein et al. (13), 2011	Age of onset of polycystic ovarian syndrome in girls may be earlier than previously thought	Yes	Assessment	Didn't include quality assessment
14.	1754	Cahill (14), 2009	PCOS	Yes		Didn't include search terms or number of articles extracted on search
15.	1755	Cahill and ***O'Brien*** (15), 2015	Polycystic ovary syndrome (PCOS): metformin	Yes		Didn't include search terms or number of articles extracted on search
16.	1756	Cai et al. (16), 2014	Association between fat mass- and obesity-associated (FTO) gene polymorphism and polycystic ovary syndrome: a meta-analysis	Yes		Didn't include quality assessment

17.	1758	Carlus et al. (17), 2016	Is MTHFR 677 C>T Polymorphism Clinically Important in Polycystic Ovarian Syndrome (PCOS)? A Case-Control Study, Meta-Analysis and Trial Sequential Analysis	Yes		Didn't include quality assessment
18.	1763	Chen et al. (18), 2014	Two follicle-stimulating hormone receptor polymorphisms and polycystic ovary syndrome risk: a meta-analysis	Yes		Didn't include quality assessment
19.	1766	Chittenden et al. (19), 2009	Polycystic ovary syndrome and the risk of gynaecological cancer: a systematic review	Yes	Assessment	Didn't include quality assessment
20.	1341	Conte et al. (20), 2015	Mental Health and Physical Activity in Women with Polycystic Ovary Syndrome: A Brief Review	Yes		Didn't include quality assessment
21.	1466	Costello et al. (21), 2010	Insulin-sensitising drugs versus the combined oral contraceptive pill for hirsutism, acne and risk of diabetes, cardiovascular disease, and endometrial cancer in polycystic ovary syndrome	Yes	Non-fertility medical treatment	No update of search strategy since 2006
22.	1478	Dissemination ***(22***)	Overweight in polycystic ovary syndrome. An update on evidence based advice on diet, exercise and metformin use for weight loss	Yes		Didn't include number of articles extracted on search or quality assessment
23.	1481	Dissemination (23)	N-acetylcysteine for treating women with clomiphene citrate resistant polycystic ovary syndrome: a systematic review	Yes		Not in English
24.	1525	Dissemination (24)	Rosiglitazone versus metformin for polycystic ovary syndrome: a systematic review (Provisional abstract)	Yes		Not in English
25.	1491	Dissemination (25)	Efficacy of inositol in women with polycystic ovary syndrome and desire for children: systematic review and meta-analysis	Yes		Not in English
26.	1539	Dissemination (26), 2015	Letrozole for ovulation induction in women with polycystic ovarian syndrome: a systematic analysis	Yes		Not in English
27.	1518	Dissemination (27), 2015	Thiazolidinediones combined with metformin in treatment of polycystic ovary syndrome: a systematic review	Yes		Not in English
28.	1540	Dissemination (28), 2015	Laparoscopic surgery versus laparotomy for women with polycystic ovarian syndrome: a systematic review.	Yes		Not in English
29.	1786	***Dokras et al. (29),*** 2011	Increased risk for abnormal depression scores in women with polycystic ovary syndrome: a systematic review and meta-analysis	Yes	Assessment	Didn't include quality assessment
30.	1794	Du et al. (30), 2010	Two FSHR variants, haplotypes and meta-analysis in Chinese women with premature ovarian failure and polycystic ovary syndrome	Yes		Didn't include search terms, quality assessment or number of articles extracted on search
31.	1793	Du and Li (31), 2013	The relationship between thyroiditis and polycystic ovary syndrome: a meta-analysis	Yes	Assessment	Didn't include quality assessment
32.	1799	Eckmann and ***Kockler (32),*** 2009	Aromatase inhibitors for ovulation and pregnancy in polycystic ovary syndrome	Yes	Fertility treatment	Didn't include number of articles extracted on search or quality assessment
33.	1804	Escobar-Morreale et al. (33), 2011	Circulating inflammatory markers in polycystic ovary syndrome: a systematic review and metaanalysis	Yes	Assessment	Didn't include quality assessment
34.	1323	Escobar-Morreale and ***Roldán-Martín*** (34), 2016	Type 1 Diabetes and Polycystic Ovary Syndrome: Systematic Review and Meta-analysis	Yes		did not address PCOS co-morbidities
35.	1806	Eyvazzadeh (35), 2009	The role of the endogenous opioid system in polycystic ovary syndrome	Yes		Not an original systematic review or meta-analysis
36.	1807	Fan et al. (36), 2013	Association between the (TAAAA)n SHBG polymorphism and PCOS: a systematic review and meta-analysis	Yes		Didn't include quality assessment

Appendix 2: Continued

37.	1572	Farquhar et al. (37), 2009	Laparoscopic ovarian diathermy versus metformin for women with polycystic ovarian syndrome	Yes	Protocol only, no review	Protocol only
38.	1818	Fernandez et al. (38), 2011	Ovarian drilling for surgical treatment of polycystic ovarian syndrome: a comprehensive review	Yes	Fertility treatment	Didn't include quality assessment
39.	1825	Frary et al. (39), 2016	The effect of dietary carbohydrates in women with polycystic ovary syndrome: a systematic review.	Yes	Lifestyle treatment	Didn't include quality assessment
40.	1826	Fu et al. (40), 2014	Association of methylenetetrahydrofolate reductase gene C677T polymorphism with polycystic ovary syndrome risk: a systematic review and meta-analysis update	Yes		Didn't include quality assessment
41.	1832	Galazis et al. (41), 2012	Proteomic biomarkers for ovarian cancer risk in women with polycystic ovary syndrome: a systematic review and biomarker database integration	Yes		Exclude, PCOS SR component update of Atiomo 2008/9 with no quality assessment
42.	1831	Galazis et al. (42), 2012	Metabolomic biomarkers of impaired glucose tolerance and type 2 diabetes mellitus with a potential for risk stratification in women with polycystic ovary syndrome	Yes		Not primarily focused on PCOS
43.	1833	Galazis et al. (43), 2013	Proteomic biomarkers of endometrial cancer risk in women with polycystic ovary syndrome: a systematic review and biomarker database integration	Yes		Exclude, PCOS SR component update of Atiomo 2008/9 with no quality assessment
44.	1829	Galazis et al. (44), 2013	Proteomic biomarkers of preterm birth risk in women with polycystic ovary syndrome (PCOS): a systematic review and biomarker database integration	Yes		Exclude, PCOS SR component update of Atiomo 2008/9 with no quality assessment
45.	1834	Gao et al. (45), 2012	Association of the T45G and G276T polymorphisms in the adiponectin gene with PCOS: A meta-analysis	Yes		Didn't include quality assessment or number of articles extracted on search
46.	2180	Garg and ***Merhi (46),*** 2016	Relationship between advanced glycation end products and steroidogenesis in PCOS.	Yes		Not an original systematic review or meta-analysis
47.	2188	Genazzani (47), 2016	Inositol as putative integrative treatment for PCOS	Yes		Not an original systematic review or meta-analysis
48.	1404	Groth (48), 2010	Adiponectin and Polycystic Ovary Syndrome	Yes	Assessment	Didn't include number of articles extracted on search or quality assessment
49.	2186	Gu et al. (49), 2016	The association between paraoxonase 1 gene polymorphisms and polycystic ovarian syndrome.	Yes	Genetics	Didn't include quality assessment or number of articles extracted on search
50.	1855	Haoula et al. (50), 2012	Evaluating the association between endometrial cancer and polycystic ovary syndrome	Yes	Assessment	Didn't include quality assessment
51.	1864	He et al. (51), 2012	A meta-analysis on the association between PPAR-gamma Pro12Ala polymorphism and polycystic ovary syndrome	Yes		Didn't include quality assessment
52.	1873	Huang et al. (52), 2012	Four polymorphisms of the CAPN 10 gene and their relationship to polycystic ovary syndrome susceptibility: a meta-analysis	Yes		Didn't include quality assessment or number of articles extracted on search
53.	1883	Ioannidis et al. (53), 2010	Polymorphisms of the insulin receptor and the insulin receptor substrates genes in polycystic ovary syndrome: a Mendelian randomization meta-analysis	Yes		Didn't include quality assessment or number of articles extracted on search
54.	1886	Jalilian et al. (54), 2015	Prevalence of polycystic ovary syndrome and its associated complications in Iranian women: A meta-analysis	Yes	Assessment	Didn't include quality assessment
55.	1395	Janci et al. (55), 2012	Polycystic Ovarian Syndrome: Metformin or Thiazolidinediones for Cardiovascular Risk Reduction?	Yes		Didn't include number of articles extracted on search or quality assessment

56.	1888	Jia et al. (56), 2013	Association of angiotensin-converting enzyme gene insertion/deletion polymorphism with polycystic ovary syndrome: a meta-analysis	Yes		Didn't include quality assessment
57.	1890	Jia et al. (57), 2014	Association between retinol-binding protein 4 and polycystic ovary syndrome: a meta-analysis	Yes	Assessment	Didn't include quality assessment
58.	1893	Johnson (58), 2011	Metformin is a reasonable first-line treatment option for non-obese women with infertility related to anovulatory polycystic ovary syndrome--a meta-analysis of randomised trials	Yes		Didn't include search terms, number of articles extracted on search or quality assessment
59.	1902	Kelly et al. (59), 2011	Insulin-like growth factor binding protein-1 in PCOS: a systematic review and meta-analysis	Yes	Assessment	Didn't include quality assessment
60.	1905	Khan et al. (60), 2015	Overlap of proteomics biomarkers between women with pre-eclampsia and PCOS: a systematic review and biomarker database integration	Yes		Exclude, PCOS SR component update of Atiomo 2008/9 with no quality assessment
61.	1907	Kjerulff et al. (61), 2011	Pregnancy outcomes in women with polycystic ovary syndrome: a metaanalysis	Yes	Assessment	Didn't include quality assessment
62.	1909	Kong et al. (62), 2015	Impact of Treatment with Metformin on Adipocytokines in Patients with Polycystic Ovary Syndrome: A Meta-Analysis	Yes	Non-fertility medical treatment	Didn't include quality assessment
63.	1913	Krul-Poel et al. (63), 2013	The role of vitamin D in metabolic disturbances in polycystic ovary syndrome: a systematic review	Yes	Assessment	Didn't include quality assessment
64.	1384	Lai et al. (64), 2014	Chinese Herbal Medicine for Oligomenorrhoea and Amenorrhoea in Polycystic Ovary Syndrome: A Systematic Review and Meta-Analysis	Yes	Complementary therapy	Abstract only
65.	1915	Lakkakula et al. (65), 2013	Genetic variants associated with insulin signaling and glucose homeostasis in the pathogenesis of insulin resistance in polycystic ovary syndrome: a systematic review	Yes		Didn't include search terms, quality assessment or number of articles extracted on search
66.	1917	Lautatzis et al. (66), 2013	Efficacy and safety of metformin during pregnancy in women with gestational diabetes mellitus or polycystic ovary syndrome: a systematic review	Yes	Non-fertility medical treatment	Didn't include search terms
67.	1918	Lee and ***Song (67),*** 2014	Plasminogen activator inhibitor-1 4G/5G and the MTHFR 677C/T polymorphisms and susceptibility to polycystic ovary syndrome: a meta-analysis	Yes		Didn't include quality assessment or number of articles extracted on search
68.	1937	Lim and ***Wong (68),*** 2010	Current evidence of acupuncture on polycystic ovarian syndrome.	Yes	Complementary therapy	Didn't include search terms, number of articles extracted on search or quality assessment
69.	1943	Lin et al. (69), 2013	Androgen receptor gene polymorphism and polycystic ovary syndrome	Yes		Didn't include quality assessment
70.	1942	Lin et al. (70), 2014	Is a GnRH antagonist protocol better in PCOS patients? A meta-analysis of RCTs	Yes	Fertility treatment	Didn't include quality assessment
71.	1946	Liu et al. (71), 2014	Plasminogen activator inhibitor-1 -675 4G/5G polymorphism and polycystic ovary syndrome risk: a meta analysis	Yes		Didn't include quality assessment
72.	1945	Liu et al. (72), 2016	Meta-analysis of the correlation between the TNF-alpha308G/A polymorphism and polycystic ovary syndrome	Yes		Didn't include quality assessment
73.	2181	Liu et al. (73), 2017	Association between fat mass and obesity associated (FTO) gene rs9939609 A/T polymorphism and polycystic ovary syndrome: a systematic review and meta-analysis.	Yes		Didn't include quality assessment
74.	1385	Louwers et al. (74), 2013	Cross-ethnic meta-analysis of genetic variants for polycystic ovary syndrome	Yes		Didn't include quality assessment or number of articles extracted on search

75.	1956	Mancini et al. (75), 2011	Gonadotrophin-releasing hormone-antagonists vs long agonist in in-vitro fertilization patients with polycystic ovary syndrome: a meta-analysis	Yes	Fertility treatment	Didn't include quality assessment
76.	1966	Misso et al. (76), 2012	Status of clomiphene citrate and metformin for infertility in PCOS	Yes		Not an original systematic review or meta-analysis
77.	1965	Misso and ***Teede (77),*** 2015	Metformin in women with PCOS, cons	Yes		Not an original systematic review or meta-analysis
78.	1978	Morris et al. (78), 2016	What does a diagnostic label of 'polycystic ovary syndrome' really mean in adolescence? A review of current practice recommendations	Yes	Assessment	Didn't include quality assessment
79.	1985	Naderpoor et al. (79), 2016	Metformin and lifestyle modification in polycystic ovary syndrome: systematic review and meta-analysis	Yes		Didn't include search terms
80.	1642	Nahuis et al. (80), 2011	Metformin co-administration during follicle stimulating hormone ovulation induction with timed intercourse or intra-uterine insemination for subfertility associated with polycystic ovary syndrome	Yes	Protocol only, no review	Protocol only
81.	1986	Nahuis et al. (81), 2013	The basic fertility workup in women with polycystic ovary syndrome: a systematic review	Yes	Assessment	Didn't include quality assessment
82.	1988	Niafar et al. (82), 2016	A systematic review of GLP-1 agonists on the metabolic syndrome in women with polycystic ovaries	Yes		Didn't include quality assessment
83.	1990	Nicholson et al. (83), 2010	Effectiveness of long-term (twelve months) nonsurgical weight loss interventions for obese women with polycystic ovary syndrome: a systematic review	Yes	Lifestyle treatment	Didn't include quality assessment
84.	1335	Palomba et al. (84), 2015	Pregnancy complications in women with polycystic ovary syndrome	Yes	Assessment	Didn't include number of articles extracted on search or quality assessment
85.	1407	Parsanezhad et al. (85), 2009	Surgical ovulation induction in women with polycystic ovary syndrome: a sytematic review	Yes	Fertility treatment	Didn't include number of articles extracted on search or quality assessment
86.	2187	Paul et al. (86), 2016	Inositol's and other nutraceuticals' synergistic actions counteract insulin resistance in polycystic ovarian syndrome and metabolic syndome: state-of-art and future perspectives.	Yes		Not an original systematic review or meta-analysis
87.	2012	Peitsidis and ***Agrawal (87),*** 2010	Role of vascular endothelial growth factor in women with PCO and PCOS: a systematic review	Yes	Assessment	Didn't include quality assessment
88.	2013	Peng et al. (88), 2014	The association between androgen receptor gene CAG polymorphism and polycystic ovary syndrome: a case-control study and meta-analysis	Yes		Didn't include quality assessment
89.	2023	Qin et al. (89), 2013	Obstetric complications in women with polycystic ovary syndrome: a systematic review and meta-analysis	Yes	Assessment	Didn't include quality assessment
90.	2025	Rajender et al. (90), 2013	Androgen receptor CAG repeats length polymorphism and the risk of polycystic ovarian syndrome (PCOS)	Yes		Didn't include quality assessment
91.	2183	Reis et al. (91), 2017	Vitamin D receptor polymorphisms and the polycystic ovary syndrome: A systematic review.	Yes		Not an original systematic review or meta-analysis
92.	2031	Ren et al. (92), 2014	[A meta-analysis on acupuncture treatment of polycystic ovary syndrome]	Yes	Complementary therapy	Not in English
93.	2036	Rocca et al. (93), 2015	Polycystic ovary syndrome: chemical pharmacotherapy	Yes		Not a systematic review
94.	2041	Saha et al. (94), 2013	N-acetyl cysteine in clomiphene citrate resistant polycystic ovary syndrome: A review of reported outcomes	Yes	Non-fertility medical treatment	Didn't include quality assessment

95.	2044	San-Millan and ***Escobar-Morreale*** (95), 2010	The role of genetic variation in peroxisome proliferator-activated receptors in the polycystic ovary syndrome (PCOS): an original case-control study followed by systematic review and meta-analysis of existing evidence	Yes		Didn't include quality assessment
96.	1663	Showell et al. (96), 2016	Inositol for subfertile women with polycystic ovary syndrome	Yes	Protocol only, no review	Protocol only
97.	2073	Sirmans et al. (97), 2012	Polycystic ovary syndrome and chronic inflammation: pharmacotherapeutic implications	Yes		Didn't include number of articles extracted on search or quality assessment
98.	2080	Song et al. (98), 2014	Lack of association of INS VNTR polymorphism with polycystic ovary syndrome: a meta-analysis	Yes		Didn't include quality assessment
99.	2087	Sun et al. (99), 2013	Effect of metformin on ovulation and reproductive outcomes in women with polycystic ovary syndrome: a meta-analysis of randomized controlled trials	Yes	Fertility treatment	Didn't include quality assessment
100.	2090	Taghavi et al. (100), 2015	Health-related quality of life in polycystic ovary syndrome patients: A systematic review	Yes		Not primarily focused on PCOS
101.	2097	Tang et al. (101), 2009	WITHDRAWN: Insulin-sensitising drugs for polycystic ovary syndrome	Yes	Fertility treatment/ Non-fertility medical treatment	Withdrawn from publication as error in citation
102.	1673	Tang et al. (102), 2010	Ultrasound-guided transvaginal ovarian needle drilling for clomiphene-resistant polycystic ovarian syndrome in subfertile women	Yes	Protocol only, no review	Protocol only
103.	2095	Tang et al. (103), 2012	Association of Pro12Ala polymorphism in peroxisome proliferator-activated receptor gamma with polycystic ovary syndrome: a meta-analysis	Yes		Didn't include quality assessment
104.	2101	Tang et al. (104), 2015	Insulin receptor substrate-1 (IRS-1) rs1801278G>A polymorphism is associated with polycystic ovary syndrome susceptibility: a meta-analysis	Yes		Didn't include quality assessment
105.	2184	Tang et al. (105), 2017	Circulating omentin-1 levels in women with polycystic ovary syndrome: a meta-analysis.	Yes		Didn't include quality assessment
106.	2103	Thethi et al. (106), 2015	Role of Insulin Sensitizers on Cardiovascular Risk Factors in Polycystic Ovarian Syndrome: A Meta-Analysis	Yes		"Reviewers worked independently and in duplicate to determine the methodological quality" but no details given
107.	1406	Tomlinson et al (107), 2010	Type 2 diabetes and cardiovascular disease in polycystic ovary syndrome: what are the risks and can they be reduced?	Yes		Didn't include search terms or number of articles extracted on search
108.	2110	Toulis et al. (108), 2011	Meta-analysis of cardiovascular disease risk markers in women with polycystic ovary syndrome	Yes	Assessment	Didn't include quality assessment
109.	2111	Tsikouras et al. (109), 2015	Features of Polycystic Ovary Syndrome in adolescence	Yes	Assessment	Didn't include number of articles extracted on search or quality assessment
110.	2116	Unfer et al. (110), 2012	Effects of myo-inositol in women with PCOS: a systematic review of randomized controlled trials	Yes	Non-fertility medical treatment	Didn't include quality assessment
111.	2134	Wang et al. (111), 2012	[Therapeutic effect of metformin for clomiphene-resistant infertility patients with polycystic ovary syndrome: a systematic analysis]	Yes	Fertility treatment	Not in English
112.	2136	Wang et al. (112), 2012	Negative association between androgen receptor gene CAG repeat polymorphism and polycystic ovary syndrome? A systematic review and meta-analysis	Yes		Didn't include quality assessment

113.	2132	Wang et al. (113), 2015	4G/5G polymorphism of plasminogen activator inhibitor-1 gene is associated with polycystic ovary syndrome in Chinese patients: a meta-analysis	Yes		Didn't include quality assessment or number of articles extracted on search
114.		Wang et al. (114), 2016	Clomiphene, metformin, letrozole, tamoxifen or combined clomiphene-metformin for polycystic ovary syndrome - a systematic review and individual participant data network meta-analysis.	Yes		Conference abstract only, no related publication found<br>No available online reference
115.	2146	Wild et al. (115), 2010	Assessment of cardiovascular risk and prevention of cardiovascular disease in women with the polycystic ovary syndrome: a consensus statement by the Androgen Excess and Polycystic Ovary Syndrome (AE-PCOS) Society	Yes		Not a systematic review
116.	2147	Wild et al. (116), 2011	Lipid levels in polycystic ovary syndrome: systematic review and meta-analysis	Yes	Assessment	Didn't include quality assessment
117.	2148	Wojciechowski et al. (117), 2012	Impact of FTO genotypes on BMI and weight in polycystic ovary syndrome: a systematic review and meta-analysis	Yes		Didn't include quality assessment
118.	2151	Wu et al. (118), 2016	Acupuncture for treating polycystic ovary syndrome: guidance for future randomized controlled trials	Yes	Complementary therapy	Didn't include number of articles extracted on search
119.	2152	Xian et al. (119), 2012	ADIPOQ gene polymorphisms and susceptibility to polycystic ovary syndrome: a HuGE survey and meta-analysis	Yes		Didn't include quality assessment
120.	1322	Xian et al. (120), 2016	Effects of metformin on pregnancy outcomes in women with polycystic ovary syndrome: A meta-analysis	Yes		Didn't include quality assessment
121.	2155	Xie et al. (121), 2013	Microsatellite polymorphism in the fibrillin 3 gene and susceptibility to PCOS: a case-control study and meta-analysis	Yes		Didn't include quality assessment or number of articles extracted on search
122.	2156	Xu et al. (122), 2014 [120]	Effect of metformin on serum interleukin-6 levels in polycystic ovary syndrome: a systematic review	Yes	Non-fertility medical treatment	Didn't include quality assessment
123.	2159	Yu et al. (123), 2014	Polymorphisms of pentanucleotide repeats (tttta)n in the promoter of CYP11A1 and their relationships to polycystic ovary syndrome (PCOS) risk: a meta-analysis	Yes		Didn't include quality assessment
124.	2185	Yu et al. (124), 2017	Comparative effectiveness of 9 ovulation-induction therapies in patients with clomiphene citrate-resistant polycystic ovary syndrome: a network meta-analysis	Yes		Didn't include search terms
125.	2163	Zhang et al. (125), 2012	Association between the Pro12Ala polymorphism of PPAR-gamma gene and the polycystic ovary syndrome: a meta-analysis of case-control studies	Yes		Didn't include quality assessment
126.	2168	Zhang et al. (126), 2014	The -675 4G/5G polymorphism in the PAI-1 gene may not contribute to the risk of PCOS	Yes		Didn't include quality assessment or number of articles extracted on search
127.	2166	Zhang et al. (127), 2015	Peroxisome proliferator-activated receptor gamma rs1801282 C>G polymorphism is associated with polycystic ovary syndrome susceptibility: a meta-analysis involving 7,069 subjects	Yes		Didn't include quality assessment
128.	2171	Zheng et al. (128), 2013	The efficacy of metformin in pregnant women with polycystic ovary syndrome: a meta-analysis of clinical trials	Yes	Non-fertility medical treatment	Didn't include quality assessment

Appendix 3: Characteristics of included reviews

Author and year	Country	Date assessed as up to date	Included studies	Study type	Language	Meta-analysis performed	Included studies quality	Systematic review guidelines followed	Population	PCOS diagnostic criteria	Participant number	Intervention	Comparison
Abu Hashim et al. (129), 2015	Egypt	Feb-15	12	RCT	Only English	Yes	Unclear	Yes	CC resistant PCOS	ESHRE/ASRM	1411	CC and metformin	Gns, LOD, AIs, NAC and other insulin sensitizers+CC
Al Khalifah et al. (130), 2016	Canada, Saudi Arabia, Colombia, UK	Jan-15	4	RCT	All languages	Yes	Low to very low quality	Yes	adolescents with PCOS (11-19 year old)	ESHRE/ASRM	231	Metformin (include con-intervention with pioglitazone, spironolactone, flutaminde or lifestyle)	Oral contraceptive pills
Baghdadi et al. (131), 2012	Australia, Saudi Arabia, Egypt, UK, Italy, UAE, Austria, China, France	Sep-11	15	Not stated	All languages	Yes	Unclear	No	CC resistant PCOS	ESHRE/ASRM	1784	OAT in lean patients	OAT in overweight and obese patients
Bordewijk et al. (132), 2017	Netherlands, Australia, Brazil	Jun-16	5	RCT and phase one in cross-over trials	All languages	Yes	Unclear	Yes	PCOS and anovulatory women	ESHRE/ASRM	264	FSH+metformin	FSH
Brown and Farquhar (133), 2017	New Zealand	Aug-16	28	RCT and phase one in cross-over trials	All languages	Yes	No	Yes	WHO group 2 anovulation	WHO classification of anovlation	3377	Antioestrogen± medical therapy, CC regimen A	Placebo, Antioestrogen± medical therapy, CC regimen B
Butterworth et al. (134), 2016	UK	Mar-15	6	Epidemiological studies	Not stated	No	No	Yes	PCOS before bariatric surgery	No specified criteria	264	Before bariatric surgery	After bariatric surgery
Ding et al. (135), 2016	China	Not stated	4	RCT	All languages	Yes	No	Yes	PCOS	ESHRE/ASRM	704	CC in luteal phase	CC in follicular phase
Fang et al. (136), 2017	China	Dec-15	9	RCT	English	Yes	Yes	No	PCOS	ESHRE/ASRM	502	Vitamin D	Placebo or Metformin
Farquhar et al. (137), 2012	New Zealand	May-12	25	RCT	All languages	Yes	Low-moderate	Yes	CC resistant PCOS	Clinical features, abnormal endocrine tests, ultrasonographic or visual appearance of ovaries	2304	LOD with or without OI<br>LOD in women undergoing ART<br>LOD techniques	Other medical treatment,<br>Without LOD<br>Various technique of LOD

Appendix 3: Continued

Feng et al. (138), 2015	China	Oct-14	5	Randomised and non-randomised control trials, total participants not <30	English	Yes	Unclear	No	PCOS, pregnant and took metformin to get conception	ESHRE/ASRM	929	Metformin throughout pregnancy	Placebo
Franik et al. (139), 2014	Netherlands, New Zealand	Sep-14	26	RCT and phase one in cross-over trials	All languages	Yes	Low	Yes	PCOS, reproductive age	ESHRE/ASRM	5560	AIs (alone or in conjunction with medical adjuncts) by sexual intercourse	AIs compared to each other and to other treatments
	Scotland	May-13	4	RCT	Only English	No	3 high quality, 1 poor quality	No	CC resistant PCOS, reproductive age	No specified criteria	129	Metformin	Placebo ± CC
Graff et al. (141), 2016	Brazil	May-15	9	prospective studies and RCT	All languages	Yes	Unclear	No	PCOS	ESHRE/ASRM	602	Orlistat for at least 8 weeks	Placebo<br>Metformin<br>Anti-obesity drugs
He and Jiang (142), 2011	China	Jun-10	6	RCT	All languages	Yes	Unclear	No	PCOS	ESHRE/ASRM	841	Letrozole	CC
Huang et al. (143), 2015	USA, China	Dec-13	12	RCT	Only English	Yes	Unclear	No	PCOS undergoing IVF/ICSI in non-donor cycles	ESHRE/ASRM	1731	Metformin	Placebo
Kollman et al. (144), 2016	Austria, UK, Brazil	Jul-15	66	RCT	English	Yes	Yes	No	PCOS	No specified criteria	6377	Any interventions aimed at improving the effectiveness of or reducing complications of ART	Not applicable
Li et al. (145), 2011	China	May-10	10	RCT	Only English	Yes	Unclear	No	PCOS	No specified criteria	459	Metformin	Thiazolidinediones
Luo et al. (146), 2014	China	Not stated	2	Not stated	Only English	Yes	Unclear	No	PCOS undergoing COS/IUI	No specified criteria	Only n. of cycles 333	GNRH antagonist+IUI	IUI
Misso et al. (147), 2012	Australia	Jul-11	13	RCT, SR of RCT	Only English	Yes	Low-high	No	PCOS	ESHRE/ASRM	2059	AIs	Placebo, no other treatment or other infertility treatment including AIs in combination with other treatment

Appendix 3: Continued

Misso et al. (148), 2013	Australia	Jul-11	4	RCT, SR of RCT	Only English	Yes	Low-Moderate	No	PCOS	ESHRE/ASRM	457	At least 1000 mg of any type of metformin at any frequency including slow release and standard release	Any type, dose and frequency of CC
Moazami et al. (149), 2014	Iran	Unclear, no time limit	6	RCT	Not stated	Yes	Unclear	Yes	CC-resistant PCOS	No specified criteria	499	LOD	Gns
Palomba et al. (150), 2009	Italy, USA	Mar-08	4	RCT	All languages	Yes	Unclear	No	PCOS	ESHRE/ASRM	1066	CC<br>CC + metformin<br>CC + metformin	Metformin<br>CC<br>metformin
Palomba et al. (151), 2009	Italy	Jun-08	17	RCT	All languages	Yes	Unclear	Yes	PCOS	ESHRE/ASRM or any non-validated criteria	1741	Pregestational metformin	Unclear
Palomba et al. (152), 2013	Italy	Aug-12	10	RCT and phase one cross over trials	All languages	Yes	Unclear	Yes	PCOS undergoing IVF cycles	No specified criteria	845	Metformin+Gns	No treatment/ placebo+ Gns
Palomba et al. (153), 2014	Italy	Oct-13	7	RCT and phase one cross over trials	All languages	Yes	Low	Yes	PCOS	No specified criteria	1023 cycles in 334 patients	Metformin	Placebo/no treatment
Pundir et al. (154), 2012	UK	Aug-10	9	RCT	All languages	Yes	Unclear	No	PCOS undergoing IVF with or without ICSI	No specified criteria	966	GNRH antagonist	GNRH agonist
Pundir et al. (155), 2017	UK, Australia	Aug-16	10	RCT	All languages	Yes	No	No	PCOS	No specified criteria	601	Inositol	Placebo Metformin Clomiphene Another isomeric form of inositol
Raval et al. (156), 2011	India, Australia	Jul-11	4	RCT and phase one cross over trials	All languages	Yes	Unclear	Yes	PCOS (not trying to conceive)	ESHRE/ASRM	244	Statins ± other drugs	Placebo<br>Other drugs
Roque et al. (157), 2015	Brazil	Oct-14	7	RCT	Only English	Yes	Unclear	Yes	PCOS (therapy naïve)	ESHRE/ASRM	1833	Letrozole	CC
Siebert et al. (158), 2012	South Africa	Nov-10	14	RCT	All languages	Yes	Unclear	No	PCOS (therapy naïve)	ESHRE/ASRM	2240	Metformin (500-2000 mg/day)	CC (CC max. 200 mg/day) or CC+Metformin
Sinawat et al. (159), 2012	Thailand	Feb-12	0	RCT	All languages	No	No studies were identified	Yes	PCOS and of reproductive age	ESHRE/ASRM	0	Short-course (less than four weeks) metformin + CC	Long-course (four weeks or more) metformin + CC.

Appendix 3: Continued

Siristatidis et al. (160), 2013	Greece, UK	May-13	0	RCT and phase one cross over trials	All languages	No	No studies were identified	Yes	PCOS	ESHRE/ASRM	0	IVM followed by IVF or ICSI. Embryo transfers of both fresh and frozen but not mixed embryos	Conventional IVF or ICSI after controlled ovarian hyper-stimulation
Siristatidis et al. (161), 2015	Greece	Oct-13	11	All study design except case series, case reports, in vitro and animal studies, narrative or systematic reviews	All languages	Yes	Unclear	Yes	PCOS, PCO and control undergoing IVM	PCOS (according to the criteria that each study adopted), PCO (ultrasonographic appearance of polycystic ovaries, as a rule) and controls (sub-fertile patients with other causes of sub-fertility, such as tubal or male factor	808 (268 PCOS patients (328 cycles), 100 PCO patients (110 cycles) and 440 controls (480 cycles))	IVM in PCOS	IVM in PCO/control
Tang et al. (162), 2012	UK, Australia	Oct-11	44	RCT and phase one cross over trials	All languages	Yes	Low	Yes	PCOS	ESHRE/ASRM	3992	Metformin, rosiglitazone or pioglitazone± ovulation induction agent	Placebo, no treatment or ovulation induction agent, CC
Thakker et al. (163), 2015	India, Usa	Sep-13	8	RCT and phase one cross over trials	All languages	Yes	Unclear	No	PCOS	ESHRE/ASRM	910	NAC ± other agent	Placebo± other agent
Tso et al. (164), 2014	Brazil, Australia	Oct-14	9	RCT and phase one cross over trials	All languages	Yes	Low-moderate	Yes	PCOS and of reproductive age undergoing IVF or ICSI	ESHRE/ASRM (Rotterdam criteria)	816	Metformin before or during IVF or ICSI treatment.	No treatment or placebo
Weiss et al. (165), 2015	Netherlands, Australia	Oct-14	14	RCT	All languages	Yes	Very low-low	Yes	CC-resistant ± failure PCOS<br>Women treated in the past by metformin with or without CC<br>Women with prior treatment with electrocautery of the ovaries.	No specified criteria	1726	OI with rFSH or FSH-HP; HMG, HP-HMG (IUI could be included)	OI with HMG, FSH-P, FSH-HP or FSH-P or FSH-P, FSH-HP (IUI could be included)

Appendix 3: Continued

Xiao et al. (166), 2012	China	Jun-11	8	RCT	English and Chinese	Yes	Little possibilities of bias - 2, moderate bias-5, high possibilities of bias -1	No	PCOS, <35 years	ESHRE/ASRM	1487	Metformin± CC	CC
Xiao et al. (167), 2013	China	Dec-11	7	RCT	English and Chinese	Yes	Insufficient quality	No	PCOS	ESHRE/ASRM	755	GNRH antagonist	GNRH agonist
Xingrong et al. (168), 2016	China, USA	Jul-13	11	not stated	All languages	Yes	Unclear	Yes	PCOS and pregnant	Not specified	1496	Metformin throughout pregnancy	No metformin during pregnancy
Zeng et al. (169), 2016	China	Sep-15	13	Any study with a control group	All languages	Yes	Unclear	No	PCOS and pregnant	ESHRE/ASRM	1606	Metformin at least 1 trimester	Placebo
Zhang et al. (170), 2014	China	Sep-13	6	RCT	English	Yes	Poor quality	No	PCOS	ESHRE/ASRM	263	Acarbose± ovulation inducing agent	Placebo, metformin
Zhuang et al. (171), 2013	China	Jun-12	1	RCT and phase one cross over trials	All language	No	Limited quality	Yes	PCOS	ESHRE/ASRM	16	Antidepressants (either alone, or in combination with other drug)	Placebo Oral contraceptive pills Metformin Lifestyle Antiobesity drugs Surgery Another antidepressant Different doses of the same antidepressant
Zhuo et al. (172) 2014	China	Dec-13	13	Any study with a control group	All languages	Yes	Unclear	No	PCOS and pregnant	ESHRE/ASRM	1828	Metformin throughout pregnancy	Placebo

AIs; Aromatase inhibitors, CC; Clomiphene citrate, COS; Controlled ovarian stimulation, ESHRE/ASRM; European society of human reproduction and embryology/American society for reproductive medicine, FSH: Follicle stimulating hormone, FSH-HP; Follicle stimulating hormone-highly purified, FSH-P; Follicle stimulating hormone-purified, GDM; Gestational diabetes mellitus, Gns; Gonadotrophins, HCG; Human chorionic gonadotrophin, HMG; Human menopausal gonadotrophin, HP-HMG; Highly purified human menopausal gonadotrophin, ICSI; Intra cytoplasmic sperm injection, IUGR; Intra-uterine growth restriction, IUI; Intra uterine insemination, IVF; In vitro fertilization, IVM; In vitro maturation, LOD; Laparoscopic ovarian drilling, NAC; N-acetyl cysteine, OAT; Ovarian ablation therapy, OHSS; Ovarian hyper-stimulation syndrome, OI; Ovulation induction, PCO; Polycystic ovary, PCOS; Polycystic ovary syndrome, PIH/PE; Pregnancy induced hypertension/Preeclampsia, RCT; Randomized control trial, rFSH; Recombinant follicle stimulating hormone, SR; Systematic review, uFSH; Urinary follicle stimulating hormone, and WHO; World Health Organization.

Appendix 4: Assessment of methodological quality of included reviews

	1 Pre-specified questions and inclusion criteria	2 Duplicate study selection and data extraction	3 Comprehensive literature search	4 Grey literature included	5 Lists included and excluded studies	6 Characteristics of included studied	7 Study quality assessed	8 Quality assessments in conclusion	9 Assessment of homogeneity	10 Assessment of publication bias	11 Conflict of interest	AMSTAR Score	Quality
Abu Hashim et al. (129), 2015	No	Yes	No	No	Yes	Yes	Yes	Yes	Yes	Yes	No	7	Moderate
Al Khalifah et al. (130), 2016	Yes	Yes	Yes	Yes	Yes	Yes	Yes	Yes	No	Yes	No	9	High
Baghdadi et al. (131), 2012	No	No	No	No	No	Yes	Yes	No	Yes	Yes	No	4	Moderate
Bordewijk et al. (132), 2017	Yes	Yes	Yes	Yes	Yes	Yes	Yes	Yes	Yes	No	No	9	High
Brown and Farquhar (133), 2017	Yes	No	Yes	Yes	Yes	Yes	Yes	Yes	Yes	Yes	No	9	High
Butterworth et al. (134), 2016	No	No	No	No	No	Yes	No	No	N/A	No	No	1	Low
Ding et al. (135), 2016	No	No	No	No	No	Yes	No	No	Yes	No	No	2	Low
Fang et al. (136), 2017	No	Yes	No	No	No	Yes	Yes	No	Yes	Yes	No	5	Moderate
Farquhar et al. (137), 2012	Yes	Yes	Yes	Yes	Yes	Yes	Yes	No	Yes	Yes	No	9	High
Feng et al. (138), 2015	No	No	No	No	No	Yes	No	No	Yes	Yes	No	3	Low
Franik et al. (139),2014	Yes	Yes	Yes	Yes	Yes	Yes	Yes	Yes	Yes	Yes	No	10	High
Dissemination (140)	Yes	No	No	No	No	Yes	Yes	N/A	N/A	N/A	No	3	Low
Graff et al. (141) 2016	Yes	Yes	No	No	No	Yes	No	No	Yes	Yes	No	5	Moderate
He and Jiang (142), 2011	No	No	No	No	No	Yes	Yes	Yes	Yes	No	No	4	Moderate
Huang et al. (143)2015	No	Yes	Yes	No	No	Yes	Yes	Yes	Yes	No	No	6	Moderate
Kollman et al. (144), 2016	Yes	Yes	Yes	No	No	Yes	Yes	Yes	Yes	Yes	No	8	High
Li et al. (145), 2011	No	No	No	No	No	Yes	Yes	Yes	Yes	No	No	4	Moderate
Luo et al. (146), 2014	No	Yes	No	No	Yes	Yes	Yes	Yes	Yes	No	No	6	Moderate
Misso et al. (147), 2012	Yes	No	Yes	No	Yes	Yes	Yes	Yes	Yes	No	Yes	8	High
Misso et al. (148), 2013	Yes	No	Yes	No	Yes	Yes	Yes	Yes	Yes	No	Yes	8	High
Moazami et al. (149), 2014	No	Yes	No	No	Yes	Yes	Yes	No	Yes	No	No	5	Moderate
Palomba et al. (150), 2009	No	No	Yes	Yes	Yes	Yes	Yes	Yes	Yes	No	No	7	Moderate
Palomba et al (151), 2009	No	No	Yes	No	Yes	Yes	Yes	Yes	Yes	No	No	6	Moderate
Palomba et al. (152), 2013	Yes	No	Yes	Yes	Yes	Yes	Yes	No	Yes	No	No	7	Moderate
Palomba et al. (153), 2014	Yes	No	Yes	Yes	No	Yes	Yes	Yes	Yes	No	No	7	Moderate
Pundir et al. (154), 2012	No	Yes	Yes	Yes	Yes	Yes	Yes	No	Yes	No	No	7	Moderate
Pundir et al. (155), 2017	No	No	Yes	Yes	Yes	Yes	Yes	No	Yes	Yes	No	7	Moderate
Raval et al. (156), 2011	Yes	Yes	Yes	Yes	Yes	Yes	Yes	No	Yes	Yes	Yes	10	High
Roque et al. (157), 2015	No	Yes	No	No	Yes	Yes	Yes	No	Yes	No	No	5	Moderate
Siebert et al. (158), 2012	No	No	Yes	No	No	Yes	No	No	Yes	No	No	3	Low
Sinawat et al. (159), 2012	Yes	No	Yes	Yes	Yes	N/A	Yes	N/A	N/A	Yes	Yes	7	Moderate
Siristatidis et al. (160), 2013	Yes	Yes	Yes	Yes	Yes	N/A	Yes	N/A	N/A	Yes	Yes	8	High
Siristatidis et al. (161), 2015	No	Yes	No	No	Yes	Yes	Yes	No	Yes	Yes	No	6	Moderate
Tang et al. (162), 2012	Yes	No	Yes	Yes	Yes	Yes	Yes	No	Yes	Yes	No	8	High
Thakker et al. (163), 2015	Yes	No	Yes	Yes	No	Yes	Yes	Yes	No	Yes	Yes	8	High
Tso et al (164), 2014	Yes	Yes	Yes	Yes	Yes	Yes	Yes	No	Yes	Yes	No	9	High
Weiss et al. (165), 2015	Yes	Yes	Yes	Yes	Yes	Yes	Yes	Yes	Yes	No	Yes	10	High
Xiao et al. (166), 2012	No	No	Yes	No	No	No	Yes	No	Yes	No	No	3	Low
Xiao et al. (167), 2013	No	No	Yes	No	No	No	Yes	Yes	Yes	No	No	4	Moderate
Xingrong et al. (168), 2016	No	No	No	No	No	Yes	No	No	Yes	Yes	No	3	Low
Zeng et al. (169), 2016	No	No	Yes	Yes	No	Yes	No	No	Yes	Yes	No	5	Moderate
Zhang et al. (170), 2014	No	No	No	No	No	Yes	Yes	Yes	Yes	Yes	No	5	Moderate
Zhuang et al. (171), 2013	Yes	Yes	Yes	Yes	Yes	Yes	Yes	No	N/a	Yes	No	8	High
Zhuo et al. (172), 2014	No	No	Yes	Yes	No	Yes	Yes	No	Yes	No	No	5	Moderate

References

1. Abu Hashim H. Twenty years of ovulation induction with metformin for PCOS; what is the best available evidence? Reprod Biomed Online. 2016; 32(1): 44-53.
2. Al Khalifah RA, Florez ID, Dennis B, Neupane B, Thabane L, Bassilious E. The effectiveness and safety of treatments used for polycystic ovarian syndrome management in adolescents: a systematic review and network meta-analysis protocol. Syst. 2015; 4: 125.
3. Al-Khalifah RA, Florez ID, Dennis B. The effectiveness and safety of treatments used for polycystic ovarian syndrome management in adolescents: a systematic review and network meta-analysis. Endocrine reviews: Conference: 98th annual meeting and expo of the endocrine society. 2016; ENDO 37.
4. Andrade C. Major malformation risk, pregnancy outcomes, and neurodevelopmental outcomes associated with metformin use during pregnancy. J Clin Psychiatry. 2016; 77(4): e411-e414.
5. Atiomo W, Khalid S, Parameshweran S, Houda M, Layfield R. Proteomic biomarkers for the diagnosis and risk stratification of polycystic ovary syndrome: a systematic review. Bjog. 2009; 116(2): 137-143.
6. Bagos PG. Plasminogen activator inhibitor-1 4G/5G and 5,10-methylene-tetrahydrofolate reductase C677T polymorphisms in polycystic ovary syndrome. Mol Hum Reprod. 2009; 15(1): 19-26.
7. Bao S, Cai JH, Yang SY, Ren Y, Feng T, Jin T, et al. Association of DENND1A gene polymorphisms with polycystic ovary syndrome: a meta-analysis. J Clin Res Pediatr Endocrinol. 2016; 8(2): 135-143.
8. Baranova A, Tran TP, Birerdinc A, Younossi ZM. Systematic review: association of polycystic ovary syndrome with metabolic syndrome and non-alcoholic fatty liver disease. Aliment Pharmacol Ther. 2011; 33(7): 801-814.
9. Barba M, Schunemann HJ, Sperati F, Akl EA, Musicco F, Guyatt G, et al. The effects of metformin on endogenous androgens and SHBG in women: a systematic review and meta-analysis. Clin Endocrinol (Oxf). 2009; 70(5): 661-670.
10. Bayram N, van Wely M, Van der Veen F. Pulsatile gonadotrophin releasing hormone for ovulation induction in subfertility associated with polycystic ovary syndrome. Cochrane Database Syst Rev. 2010; (11).
11. Birch Petersen K, Pedersen NG, Pedersen AT, Lauritsen MP, la Cour Freiesleben N. Mono-ovulation in women with polycystic ovary syndrome: a clinical review on ovulation induction. Reprod Biomed Online. 2016; 32(6): 563-583.
12. Bouza-Alvarez C, Ruiz-Lopez M, Alcazar-Alcazar R, Lopez-Cuadrado T. Safety and efficacy of metformin in improving clinical, hormonal and metabolic features of polycystic ovary syndrome. Systematic review and meta-analysis Madri. Agencia de Evaluacion de Tecnologias Sanitarias (AETS). 2011.
13. Bronstein J, Tawdekar S, Liu Y, Pawelczak M, David R, Shah B. Age of onset of polycystic ovarian syndrome in girls may be earlier than previously thought. J Pediatr Adolesc Gynecol. 2011; 24(1): 15-20.
14. Cahill D. Pcos. Clin Evid (Online). 2009.
15. Cahill DJ, O'Brien K. Polycystic ovary syndrome (PCOS): metformin. Clin Evid (Online). 2015.
16. Cai X, Liu C, Mou S. Association between fat mass- and obesity-associated (FTO) gene polymorphism and polycystic ovary syndrome: a meta-analysis. PLoS One. 2014; 9(1): e86972.
17. Carlus SJ, Sarkar S, Bansal SK, Singh V, Singh K, Jha RK, et al. Is MTHFR 677 C>T polymorphism clinically important in polycystic ovarian syndrome (PCOS)? A case-control study, meta-analysis and trial sequential analysis. PLoS One. 2016; 11(3): e0151510.
18. Chen DJ, Ding R, Cao JY, Zhai JX, Zhang JX, Ye DQ. Two follicle-stimulating hormone receptor polymorphisms and polycystic ovary syndrome risk: a meta-analysis. Eur J Obstet Gynecol Reprod Biol. 2014; 182: 27-32.
19. Chittenden BG, Fullerton G, Maheshwari A, Bhattacharya S. Polycystic ovary syndrome and the risk of gynaecological cancer: a systematic review. Reprod Biomed Online. 2009; 19(3): 398-405.
20. Conte F, Banting L, Teede H, Stepto N. Mental health and physical activity in women with polycystic ovary syndrome: a brief review. Sports Medicine. 2015; 45(4): 497-504.
21. Costello MF, Shrestha B, Eden J, Johnson N, Moran LJ. Insulin-sensitising drugs versus the combined oral contraceptive pill for hirsutism, acne and risk of diabetes, cardiovascular disease, and endometrial cancer in polycystic ovary syndrome. Cochrane Database Syst Rev. 2010; (11).
22. Dissemination CfRa. Overweight in polycystic ovary syndrome. An update on evidence based advice on diet, exercise and metformin use for weight loss (Provisional abstract). DARE. 2015; (2).
23. Dissemination CfRa. N-acetylcysteine for treating women with clomiphene citrate resistant polycystic ovary syndrome: a systematic review (Provisional abstract). DARE. 2015; (2).
24. Dissemination CfRa. Rosiglitazone versus metformin for polycystic ovary syndrome: a systematic review (Provisional abstract). DARE. 2015; (2).
25. Dissemination CfRa. Efficacy of inositol in women with polycystic ovary syndrome and desire for children: systematic review and meta-analysis (Provisional abstract). DARE. 2015; (2).
26. Dissemination CfRa. Letrozole for ovulation induction in women with polycystic ovarian syndrome: a systematic analysis (Provisional abstract). DARE. 2015; (2).
27. Dissemination CfRa. Thiazolidinediones combined with metformin in treatment of polycystic ovary syndrome: a systematic review (Provisional abstract). DARE. 2015; (2).
28. Dissemination CfRa. Laparoscopic surgery versus laparotomy for women with polycystic ovarian syndrome: a systematic review (Provisional abstract). DARE. 2015; (2).
29. Dokras A, Clifton S, Futterweit W, Wild R. Increased risk for abnormal depression scores in women with polycystic ovary syndrome: a systematic review and meta-analysis. Obstet Gynecol. 2011; 117(1): 145-152.
30. Du J, Zhang W, Guo L, Zhang Z, Shi H, Wang J, et al. Two FSHR variants, haplotypes and meta-analysis in Chinese women with premature ovarian failure and polycystic ovary syndrome. Mol Genet Metab. 2010; 100(3): 292-295.
31. Du D, Li X. The relationship between thyroiditis and polycystic ovary syndrome: a meta-analysis. Int J Clin Exp Med. 2013; 6(10): 880-889.
32. Eckmann KR, Kockler DR. Aromatase inhibitors for ovulation and pregnancy in polycystic ovary syndrome. Ann Pharmacother. 2009; 43(7): 1338-1346.
33. Escobar-Morreale HF, Luque-Ramirez M, Gonzalez F. Circulating inflammatory markers in polycystic ovary syndrome: a systematic review and metaanalysis. Fertil Steril. 2011; 95(3): 1048-58. e1-e2.
34. Escobar-Morreale HF, Roldán-Martín MB. Type 1 diabetes and polycystic ovary syndrome: systematic review and meta-analysis. Diabetes Care. 2016; 39(4): 639-648.
35. Eyvazzadeh AD, Pennington KP, Pop-Busui R, Sowers M, Zubieta JK, Smith YR. The role of the endogenous opioid system in polycystic ovary syndrome. Fertil Steril. 2009; 92(1): 1-12.
36. Fan W, Li S, Chen Q, Huang Z. Association between the (TAAAA) n SHBG polymorphism and PCOS: a systematic review and meta-analysis. Gynecol Endocrinol. 2013; 29(7): 645-650.
37. Farquhar C, Ying T, Wu T. Laparoscopic ovarian diathermy versus metformin for women with polycystic ovarian syndrome. Cochrane Database Syst Rev. 2009; (4).
38. Fernandez H, Morin-Surruca M, Torre A, Faivre E, Deffieux X, Gervaise A. Ovarian drilling for surgical treatment of polycystic ovarian syndrome: a comprehensive review. Reprod Biomed Online. 2011; 22(6): 556-568.
39. Frary JM, Bjerre KP, Glintborg D, Ravn P. The effect of dietary carbohydrates in women with polycystic ovary syndrome: a systematic review. Minerva Endocrinol. 2016; 41(1): 57-69.
40. Fu LY, Dai LM, Li XG, Zhang K, Bai Y. Association of methylenetetrahydrofolate reductase gene C677T polymorphism with polycystic ovary syndrome risk: a systematic review and meta-analysis update. Eur J Obstet Gynecol Reprod Biol. 2014; 172: 56-61.
41. Galazis N, Olaleye O, Haoula Z, Layfield R, Atiomo W. Proteomic biomarkers for ovarian cancer risk in women with polycystic ovary syndrome: a systematic review and biomarker database integration. Fertil Steril. 2012; 98(6): 1590-1601. e1.
42. Galazis N, Iacovou C, Haoula Z, Atiomo W. Metabolomic biomarkers of impaired glucose tolerance and type 2 diabetes mellitus with a potential for risk stratification in women with polycystic ovary syndrome. Eur J Obstet Gynecol Reprod Biol. 2012; 160(2): 121-130.
43. Galazis N, Pang YL, Galazi M, Haoula Z, Layfield R, Atiomo W. Proteomic biomarkers of endometrial cancer risk in women with polycystic ovary syndrome: a systematic review and biomarker database integration. Gynecol Endocrinol. 2013; 29(7): 638-644.
44. Galazis N, Docheva N, Nicolaides KH, Atiomo W. Proteomic biomarkers of preterm birth risk in women with polycystic ovary syndrome (PCOS): a systematic review and biomarker database integration. PLoS One. 2013; 8(1): e53801.
45. Gao L, Zhang Y, Cui Y, Jiang Y, Wang X, Liu J. Association of the T45G and G276T polymorphisms in the adiponectin gene with

PCOS: a meta-analysis. Gynecol Endocrinol. 2012; 28(2): 106-110.
46. Garg D, Merhi Z. Relationship between advanced glycation end products and steroidogenesis in PCOS. Reprod Biol Endocrinol. 2016; 14(1): 71.
47. Genazzani AD. Inositol as putative integrative treatment for PCOS. Reprod Biomed Online. 2016; 33(6): 770-780.
48. Groth SW. Adiponectin and polycystic ovary syndrome. Biol Res Nurs. 2010; 12(1): 62-72.
49. Gu HF, Mou M, Liang ZG, Sun C, Ren XY, Xiao YB. The association between paraoxonase 1 gene polymorphisms and polycystic ovarian syndrome. Mol Cell Biol (Noisy-le-Grand, France). 2016; 62(14): 44-47.
50. Haoula Z, Salman M, Atiomo W. Evaluating the association between endometrial cancer and polycystic ovary syndrome. Hum Reprod. 2012; 27(5): 1327-1331.
51. He J, Wang L, Liu J, Liu F, Li X. A meta-analysis on the association between PPAR-gamma Pro12Ala polymorphism and polycystic ovary syndrome. J Assist Reprod Genet. 2012; 29(7): 669-677.
52. Huang M, Xiao J, Zhao X, Liu C, Chen Q. Four polymorphisms of the CAPN 10 gene and their relationship to polycystic ovary syndrome susceptibility: a meta-analysis. Clin Endocrinol (Oxf). 2012; 76(3): 431-438.
53. Ioannidis A, Ikonomi E, Dimou NL, Douma L, Bagos PG. Polymorphisms of the insulin receptor and the insulin receptor substrates genes in polycystic ovary syndrome: a mendelian randomization meta-analysis. Mol Genet Metab. 2010; 99(2): 174-183.
54. Jalilian A, Kiani F, Sayehmiri F, Sayehmiri K, Khodaee Z, Akbari M. Prevalence of polycystic ovary syndrome and its associated complications in Iranian women: a meta-analysis. Iran J Reprod Med. 2015; 13(10): 591-604.
55. Janci MM, Smith RC, Odegard PS. Polycystic ovarian syndrome: metformin or thiazolidinediones for cardiovascular risk reduction? Diabetes Spectr. 2012; 25(4): 229-237.
56. Jia H, Wang B, Yu L, Jiang Z. Association of angiotensin-converting enzyme gene insertion/deletion polymorphism with polycystic ovary syndrome: a meta-analysis. J Renin Angiotensin Aldosterone Syst. 2013; 14(3): 255-262.
57. Jia J, Bai J, Liu Y, Yin J, Yang P, Yu S, et al. Association between retinol-binding protein 4 and polycystic ovary syndrome: a meta-analysis. Endocr J. 2014; 61(10): 995-1002.
58. Johnson N. Metformin is a reasonable first-line treatment option for non-obese women with infertility related to anovulatory polycystic ovary syndrome--a meta-analysis of randomised trials. Aust N Z J Obstet Gynaecol. 2011; 51(2): 125-129.
59. Kelly CJ, Stenton SR, Lashen H. Insulin-like growth factor binding protein-1 in PCOS: a systematic review and meta-analysis. Hum Reprod Update. 2011; 17(1): 4-16.
60. Khan GH, Galazis N, Docheva N, Layfield R, Atiomo W. Overlap of proteomics biomarkers between women with pre-eclampsia and PCOS: a systematic review and biomarker database integration. Hum Reprod. 2015; 30(1): 133-148.
61. Kjerulff LE, Sanchez-Ramos L, Duffy D. Pregnancy outcomes in women with polycystic ovary syndrome: a metaanalysis. Am J Obstet Gynecol. 2011; 204(6): 558. e1-6.
62. Kong W, Niu X, Zeng T, Lu M, Chen L. Impact of treatment with metformin on adipocytokines in patients with polycystic ovary syndrome: a meta-analysis. PLoS One. 2015; 10(10): e0140565.
63. Krul-Poel YH, Snackey C, Louwers Y, Lips P, Lambalk CB, Laven JS, et al. The role of vitamin D in metabolic disturbances in polycystic ovary syndrome: a systematic review. Eur. 2013; 169(6): 853-865.
64. Lai L, Li X, Flower A, Moore M, Liu J. Chinese herbal medicine for oligomenorrhoea and amenorrhoea in polycystic ovary syndrome: a systematic review and meta-analysis. J Altern Complement Med. 2014; 20(5): A129-A.
65. Lakkakula BV, Thangavelu M, Godla UR. Genetic variants associated with insulin signaling and glucose homeostasis in the pathogenesis of insulin resistance in polycystic ovary syndrome: a systematic review. J Assist Reprod Genet. 2013; 30(7): 883-895.
66. Lautatzis ME, Goulis DG, Vrontakis M. Efficacy and safety of metformin during pregnancy in women with gestational diabetes mellitus or polycystic ovary syndrome: a systematic review. Metabolism. 2013; 62(11): 1522-1534.
67. Lee YH, Song GG. Plasminogen activator inhibitor-1 4G/5G and the MTHFR 677C/T polymorphisms and susceptibility to polycystic ovary syndrome: a meta-analysis. Eur J Obstet Gynecol Reprod Biol. 2014; 175: 8-14.
68. Lim CE, Wong WS. Current evidence of acupuncture on polycystic ovarian syndrome. Gynecol Endocrinol. 2010; 26(6): 473-478.
69. Lin LH, Baracat MC, Maciel GA, Soares JM, Jr, Baracat EC. Androgen receptor gene polymorphism and polycystic ovary syndrome. Int J Gynaecol Obstet. 2013; 120(2): 115-118.
70. Lin H, Li Y, Li L, Wang W, Yang D, Zhang Q. Is a GnRH antagonist protocol better in PCOS patients? A meta-analysis of RCTs. PLoS One. 2014; 9(3): e91796.
71. Liu Y, Sun MG, Jiang R, Ding R, Che Z, Chen YY, et al. Plasminogen activator inhibitor-1 -675 4G/5G polymorphism and polycystic ovary syndrome risk: a meta analysis. J Assist Reprod Genet. 2014; 31(3): 363-370.
72. Liu XB, Deng XH, Zhou B, Zhang L, Niu XM. Meta-analysis of the correlation between the TNF-alpha308G/A polymorphism and polycystic ovary syndrome. Genet Mol Res. 2016; 15(2).
73. Liu AL, Xie HJ, Xie HY, Liu J, Yin J, Hu JS, et al. Association between fat mass and obesity associated (FTO) gene rs9939609 A/T polymorphism and polycystic ovary syndrome: a systematic review and meta-analysis. BMC Med Genet. 2017; 18(1): 89.
74. Louwers YV, Stolk L, Uitterlinden AG, Laven JSE. Cross-ethnic meta-analysis of genetic variants for polycystic ovary syndrome. J Clin Endocrinol Metab. 2013; 98(12): E2006-E2012.
75. Mancini F, Tur R, Martinez F, Coroleu B, Rodriguez I, Barri PN. Gonadotrophin-releasing hormone-antagonists vs long agonist in in-vitro fertilization patients with polycystic ovary syndrome: a meta-analysis. Gynecol Endocrinol. 2011; 27(3): 150-155.
76. Misso ML, Teede HJ, Hart R, Wong J, Rombauts L, Melder AM, et al. Status of clomiphene citrate and metformin for infertility in PCOS. Trends Endocrinol Metab. 2012; 23(10): 533-543.
77. Misso ML, Teede HJ. Metformin in women with PCOS, cons. Endocrine. 2015; 48(2): 428-433.
78. Morris S, Grover S, Sabin MA. What does a diagnostic label of 'polycystic ovary syndrome' really mean in adolescence? A review of current practice recommendations. Clin. 2016; 6(1): 1-18.
79. Naderpoor N, Shorakae S, de Courten B, Misso ML, Moran LJ, Teede HJ. Metformin and lifestyle modification in polycystic ovary syndrome: systematic review and meta-analysis. Hum Reprod Update. 2015; 21(5): 560-574.
80. Nahuis M, Costello MF, Van der Veen F, Tso LO, Oosterhuis J, Mol WB, et al. Metformin co-administration during follicle stimulating hormone ovulation induction with timed intercourse or intra-uterine insemination for subfertility associated with polycystic ovary syndrome. Cochrane Database Syst Rev. 2011; (5).
81. Nahuis MJ, Oosterhuis GJ, Hompes PG, van Wely M, Mol BW, van der Veen F. The basic fertility workup in women with polycystic ovary syndrome: a systematic review. Fertil Steril. 2013; 100(1):219-25.
82. Niafar M, Pourafkari L, Porhomayon J, Nader N. A systematic review of GLP-1 agonists on the metabolic syndrome in women with polycystic ovaries. Arch Gynecol Obstet. 2016; 293(3): 509-515.
83. Nicholson F, Rolland C, Broom J, Love J. Effectiveness of long-term (twelve months) nonsurgical weight loss interventions for obese women with polycystic ovary syndrome: a systematic review. Int J Women Health. 2010; 2: 393-399.
84. Palomba S, de Wilde MA, Falbo A, Koster MPH, La Sala GB, Fauser BCJM. Pregnancy complications in women with polycystic ovary syndrome. Hum Reprod Update. 2015; 21(5): 575-592.
85. Parsanezhad ME, Zarei A, Sayadi M, Jaafarzadeh A, Rajaeefard A, Frank V, et al. Surgical ovulation induction in women with polycystic ovary syndrome: a sytematic review. Iran J Med Sci. 2009; 34(4): 225-241.
86. Paul C, Lagana AS, Maniglio P, Triolo O, Brady DM. Inositol's and other nutraceuticals' synergistic actions counteract insulin resistance in polycystic ovarian syndrome and metabolic syndrome: state-of-the-art and future perspectives. Gynecol Endocrinol. 2016; 32(6): 431-438.
87. Peitsidis P, Agrawal R. Role of vascular endothelial growth factor in women with PCO and PCOS: a systematic review. Reprod Biomed Online. 2010; 20(4): 444-452.
88. Peng CY, Xie HJ, Guo ZF, Nie YL, Chen J, Zhou JM, et al. The association between androgen receptor gene CAG polymorphism and polycystic ovary syndrome: a case-control study and meta-analysis. J Assist Reprod Genet. 2014; 31(9): 1211-1219.
89. Qin JZ, Pang LH, Li MJ, Fan XJ, Huang RD, Chen HY. Obstetric complications in women with polycystic ovary syndrome: a systematic review and meta-analysis. Reprod Biol Endocrinol. 2013; 11: 56.
90. Rajender S, Carlus SJ, Bansal SK, Negi MP, Sadasivam N, Sadasivam MN, et al. Androgen receptor CAG repeats length polymorphism and the risk of polycystic ovarian syndrome (PCOS). PLoS

One. 2013; 8(10): e75709.
91. Reis GV, Gontijo NA, Rodrigues KF, Alves MT, Ferreira CN, Gomes KB. Vitamin D receptor polymorphisms and the polycystic ovary syndrome: a systematic review. J Obstet Gynaecol Res. 2017; 43(3): 436-446.
92. Ren LN, Guo LH, Ma WZ, Zhang R. A meta-analysis on acupuncture treatment of polycystic ovary syndrome. Chen Tzu Yen Chiu. 2014; 39(3): 238-246.
93. Rocca ML, Venturella R, Mocciaro R, Di Cello A, Sacchinelli A, Russo V, et al. Polycystic ovary syndrome: chemical pharmacotherapy. Expert Opin Pharmacother. 2015; 16(9): 1369-1393.
94. Saha L, Kaur S, Saha PK. N-acetyl cysteine in clomiphene citrate resistant polycystic ovary syndrome: A review of reported outcomes. J Pharmacol Pharmacother. 2013; 4(3): 187-191.
95. San-Millan JL, Escobar-Morreale HF. The role of genetic variation in peroxisome proliferator-activated receptors in the polycystic ovary syndrome (PCOS): an original case-control study followed by systematic review and meta-analysis of existing evidence. Clin Endocrinol (Oxf). 2010; 72(3): 383-392.
96. Showell MG, MackenzieProctor R, Jordan V, Hodgson R, Brown J, Farquhar C. Inositol for subfertile women with polycystic ovary syndrome. Cochrane Database Syst Rev. 2016; (9).
97. Sirmans SM, Weidman-Evans E, Everton V, Thompson D. Polycystic ovary syndrome and chronic inflammation: pharmacotherapeutic implications. Ann Pharmacother. 2012; 46(3): 403-418.
98. Song LY, Luo JR, Peng QL, Wang J, Xie L, He Y, et al. Lack of association of INS VNTR polymorphism with polycystic ovary syndrome: a meta-analysis. J Assist Reprod Genet. 2014; 31(6): 675-681.
99. Sun X, Zhang D, Zhang W. Effect of metformin on ovulation and reproductive outcomes in women with polycystic ovary syndrome: a meta-analysis of randomized controlled trials. Arch Gynecol Obstet. 2013; 288(2): 423-430.
100. Taghavi SA, Bazarganipour F, Montazeri A, Kazemnejad A, Chaman R, Khosravi A. Health-related quality of life in polycystic ovary syndrome patients: a systematic review. Iran J Reprod Med. 2015; 13(8): 473-482.
101. Tang T, Lord JM, Norman RJ, Yasmin E, Balen AH. WITHDRAWN: Insulin-sensitising drugs for polycystic ovary syndrome. Cochrane Database Syst Rev. 2009; (3): CD003053.
102. Tang L, Xu L, Pan X, Zhang J, Wu T, Liu GJ. Ultrasound-guided transvaginal ovarian needle drilling for clomiphene-resistant polycystic ovarian syndrome in subfertile women. Cochrane Database Syst Rev. 2010; (11).
103. Tang ST, Wang CJ, Tang HQ, Peng WJ, Wang YM, Zhang Q. Association of Pro12Ala polymorphism in peroxisome proliferator-activated receptor gamma with polycystic ovary syndrome: a meta-analysis. Mol Biol Rep. 2012; 39(10): 9649-9660.
104. Tang W, Wang Y, Jiang H, Liu C, Dong C, Chen S, et al. Insulin receptor substrate-1 (IRS-1) rs1801278G>A polymorphism is associated with polycystic ovary syndrome susceptibility: a meta-analysis. Int J Clin Exp Med. 2015; 8(10): 17451-17460.
105. Tang YL, Yu J, Zeng ZG, Liu Y, Liu JY, Xu JX. Circulating omentin-1 levels in women with polycystic ovary syndrome: a meta-analysis. Gynecol Endocrinol. 2017; 33(3): 244-249.
106. Thethi TK, Katalenich B, Nagireddy P, Chabbra P, Kuhadiya N, Fonseca V. Role of insulin sensitizers on cardiovascular risk factors in polycystic ovarian syndrome: a meta-analysis. Endocr Pract. 2015; 21(6): 645-667.
107. Tomlinson J, Millward A, Stenhouse E, Pinkney J. Type 2 diabetes and cardiovascular disease in polycystic ovary syndrome: what are the risks and can they be reduced? Diabet Med. 2010; 27(5): 498-515.
108. Toulis KA, Goulis DG, Mintziori G, Kintiraki E, Eukarpidis E, Mouratoglou SA, et al. Meta-analysis of cardiovascular disease risk markers in women with polycystic ovary syndrome. Hum Reprod Update. 2011; 17(6): 741-760.
109. Tsikouras P, Spyros L, Manav B, Zervoudis S, Poiana C, Nikolaos T, et al. Features of polycystic ovary syndrome in adolescence. J Med Life. 2015; 8(3): 291-296.
110. Unfer V, Carlomagno G, Dante G, Facchinetti F. Effects of myoinositol in women with PCOS: a systematic review of randomized controlled trials. Gynecol Endocrinol. 2012; 28(7): 509-515.
111. Wang LL, Ren W, Cheng QF, Fan XD. Therapeutic effect of metformin for clomiphene-resistant infertility patients with polycystic ovary syndrome: a systematic analysis. Chung Hua Fu Chan Ko Tsa Chih. 2012; 47(9): 659-663.
112. Wang R, Goodarzi MO, Xiong T, Wang D, Azziz R, Zhang H. Negative association between androgen receptor gene CAG repeat polymorphism and polycystic ovary syndrome? A systematic review and meta-analysis. Mol Hum Reprod. 2012; 18(10): 498-509.
113. Wang LH, Wang LM, Zhou N. 4G/5G polymorphism of plasminogen activator inhibitor-1 gene is associated with polycystic ovary syndrome in Chinese patients: a meta-analysis. Arch Gynecol Obstet. 2015; 292(3): 683-636.
114. Wang R, Kim BV, Zhang H, Moll E, Van Wely M, Johnson NP, et al. Clomiphene, metformin, letrozole, tamoxifen or combined clomiphene-metformin for polycystic ovary syndrome-a systematic review and individual participant data network meta-analysis. Hum Reprod Update. Conference: 32nd annual meeting of the european society of human reproduction and embryology Finland. 2016; 31: i441-i442.
115. Wild RA, Carmina E, Diamanti-Kandarakis E, Dokras A, Escobar-Morreale HF, Futterweit W, et al. Assessment of cardiovascular risk and prevention of cardiovascular disease in women with the polycystic ovary syndrome: a consensus statement by the Androgen Excess and Polycystic Ovary Syndrome (AE-PCOS) Society. J Clin Endocrinol Metab. 2010; 95(5): 2038-2049.
116. Wild RA, Rizzo M, Clifton S, Carmina E. Lipid levels in polycystic ovary syndrome: systematic review and meta-analysis. Fertil Steril. 2011; 95(3): 1073-1079. e1-11.
117. Wojciechowski P, Lipowska A, Rys P, Ewens KG, Franks S, Tan S, et al. Impact of FTO genotypes on BMI and weight in polycystic ovary syndrome: a systematic review and meta-analysis. Diabetologia. 2012; 55(10): 2636-2645.
118. Wu Y, Robinson N, Hardiman PJ, Taw MB, Zhou J, Wang FF, et al. Acupuncture for treating polycystic ovary syndrome: guidance for future randomized controlled trials. J Zhejiang Univ Sci B. 2016; 17(3): 169-180.
119. Xian L, He W, Pang F, Hu Y. ADIPOQ gene polymorphisms and susceptibility to polycystic ovary syndrome: a HuGE survey and meta-analysis. Eur J Obstet Gynecol Reprod Biol. 2012; 161(2): 117-124.
120. Xian-Ling Z, Ya-Fei Z, Quan T, Yan X, Rui-Fang A, Zeng X-L, et al. Effects of metformin on pregnancy outcomes in women with polycystic ovary syndrome: a meta-analysis. Medicine. 2016; 95(36): 1-10.
121. Xie GB, Xu P, Che YN, Xia YJ, Cao YX, Wang WJ, et al. Microsatellite polymorphism in the fibrillin 3 gene and susceptibility to PCOS: a case-control study and meta-analysis. Reprod Biomed Online. 2013; 26(2): 168-174.
122. Xu X, Du C, Zheng Q, Peng L, Sun Y. Effect of metformin on serum interleukin-6 levels in polycystic ovary syndrome: a systematic review. BMC Womens Health. 2014; 14: 93.
123. Yu M, Feng R, Sun X, Wang H, Wang H, Sang Q, et al. Polymorphisms of pentanucleotide repeats (tttta)n in the promoter of CYP11A1 and their relationships to polycystic ovary syndrome (PCOS) risk: a meta-analysis. Mol Biol Rep. 2014; 41(7): 4435-4445.
124. Yu Y, Fang L, Zhang R, He J, Xiong Y, Guo X, et al. Comparative effectiveness of 9 ovulation-induction therapies in patients with clomiphene citrate-resistant polycystic ovary syndrome: a network meta-analysis. Sci Rep. 2017; 7(1): 3812.
125. Zhang H, Bi Y, Hu C, Lu W, Zhu D. Association between the Pro12Ala polymorphism of PPAR-gamma gene and the polycystic ovary syndrome: a meta-analysis of case-control studies. Gene. 2012; 503(1): 12-17.
126. Zhang TT, Yuan L, Yang YM, Ren Y. The -675 4G/5G polymorphism in the PAI-1 gene may not contribute to the risk of PCOS. Eur Rev Med Pharmacol Sci. 2014; 18(16): 2326-2331.
127. Zhang S, Wang Y, Jiang H, Liu C, Sun B, Chen S, et al. Peroxisome proliferator-activated receptor gamma rs1801282 C>G polymorphism is associated with polycystic ovary syndrome susceptibility: a meta-analysis involving 7,069 subjects. Int J Clin Exp Med. 2015; 8(10): 17418-17429.
128. Zheng J, Shan PF, Gu W. The efficacy of metformin in pregnant women with polycystic ovary syndrome: a meta-analysis of clinical trials. J Endocrinol Invest. 2013; 36(10): 797-802.
129. Abu Hashim H, Foda O, Ghayaty E. Combined metformin-clomiphene in clomiphene-resistant polycystic ovary syndrome: a systematic review and meta-analysis of randomized controlled trials. Acta Obstet Gynecol Scand. 2015; 94(9): 921-930.
130. Al Khalifah RA, Florez ID, Dennis B, Thabane L, Bassilious E. Metformin or oral contraceptives for adolescents with polycystic ovarian syndrome: a meta-analysis. Pediatrics. 2016; 137(5): 1-12.
131. Baghdadi LR, Abu Hashim H, Amer SA, Palomba S, Falbo A, Al-Ojaimi E, et al. Impact of obesity on reproductive outcomes after ovarian ablative therapy in PCOS: a collaborative meta-analysis. Reprod Biomed Online. 2012; 25(3): 227-241.

132. Bordewijk EM, Nahuis M, Costello MF, Van der Veen F, Tso LO, Mol BW, et al. Metformin during ovulation induction with gonadotrophins followed by timed intercourse or intrauterine insemination for subfertility associated with polycystic ovary syndrome. The Cochrane database Syst Rev. 2017; 1: Cd009090.
133. Brown J, Farquhar C. Clomiphene and other antioestrogens for ovulation induction in polycystic ovarian syndrome. The Cochrane database Syst Rev. 2016; 12: Cd002249.
134. Butterworth J, Deguara J, Borg CM. Bariatric Surgery, polycystic ovary syndrome, and infertility. J Obes. 2016; 2016: 1871594.
135. Ding N, Chang J, Jian Q, Liang X, Liang Z, Wang F. Luteal phase clomiphene citrate for ovulation induction in women with polycystic ovary syndrome: a systematic review and meta-analysis. Gynecol Endocrinol. 2016; 32(11): 866-871.
136. Fang F, Ni K, Cai Y, Shang J, Zhang X, Xiong C. Effect of vitamin D supplementation on polycystic ovary syndrome: A systematic review and meta-analysis of randomized controlled trials. Complement Ther Clin Pract. 2017; 26: 53-60.
137. Farquhar C, Brown J, Marjoribanks J. Laparoscopic drilling by diathermy or laser for ovulation induction in anovulatory polycystic ovary syndrome. Cochrane Database Syst Rev. 2012; 6: CD001122.
138. Feng L, Lin XF, Wan ZH, Hu D, Du YK. Efficacy of metformin on pregnancy complications in women with polycystic ovary syndrome: a meta-analysis. Gynecol Endocrinol. 2015; 31(11): 833-839.
139. Franik S, Kremer AMJ, Nelen LDMW, Farquhar C. Aromatase inhibitors for subfertile women with polycystic ovary syndrome. Cochrane Database Syst Rev. 2014; (10).
140. Dissemination CfRa. Does metformin combined with clomiphene citrate improve fertility related outcomes in clomiphene resistant women with PCOS: a systematic review (Provisional abstract). DARE. 2015; (2).
141. Graff SK, Mario FM, Ziegelmann P, Spritzer PM. Effects of orlistat vs. metformin on weight loss-related clinical variables in women with PCOS: systematic review and meta-analysis. Int J Clin Pract. 2016; 70(6): 450-461.
142. He D, Jiang F. Meta-analysis of letrozole versus clomiphene citrate in polycystic ovary syndrome. Reprod Biomed Online. 2011; 23(1): 91-96.
143. Huang X, Wang P, Tal R, Lv F, Li Y, Zhang X. A systematic review and meta-analysis of metformin among patients with polycystic ovary syndrome undergoing assisted reproductive technology procedures. Int J Gynaecol Obstet. 2015; 131(2): 111-116.
144. Kollmann M, Martins WP, Lima ML, Craciunas L, Nastri CO, Richardson A, et al. Strategies for improving outcome of assisted reproduction in women with polycystic ovary syndrome: systematic review and meta-analysis. Ultrasound Obstet Gynecol. 2016; 48(6): 709-718.
145. Li XJ, Yu YX, Liu CQ, Zhang W, Zhang HJ, Yan B, et al. Metformin vs thiazolidinediones for treatment of clinical, hormonal and metabolic characteristics of polycystic ovary syndrome: a meta-analysis. Clin Endocrinol (Oxf). 2011; 74(3): 332-339.
146. Luo S, Li S, Li X, Bai Y, Jin S. Effect of gonadotropin-releasing hormone antagonists on intrauterine insemination cycles in women with polycystic ovary syndrome: a meta-analysis. Gynecol Endocrinol. 2014; 30(4): 255-259.
147. Misso ML, Wong JL, Teede HJ, Hart R, Rombauts L, Melder AM, et al. Aromatase inhibitors for PCOS: a systematic review and meta-analysis. Hum Reprod Update. 2012; 18(3): 301-312.
148. Misso ML, Costello MF, Garrubba M, Wong J, Hart R, Rombauts L, et al. Metformin versus clomiphene citrate for infertility in non-obese women with polycystic ovary syndrome: a systematic review and meta-analysis. Hum Reprod Update. 2013; 19(1): 2-11.
149. Moazami Goudarzi Z, Fallahzadeh H, Aflatoonian A, Mirzaei M. Laparoscopic ovarian electrocautery versus gonadotropin therapy in infertile women with clomiphene citrate-resistant polycystic ovary syndrome: A systematic review and meta-analysis. Iran J Reprod Med. 2014; 12(8): 531-538.
150. Palomba S, Pasquali R, Orio F, Jr., Nestler JE. Clomiphene citrate, metformin or both as first-step approach in treating anovulatory infertility in patients with polycystic ovary syndrome (PCOS): a systematic review of head-to-head randomized controlled studies and meta-analysis. Clin Endocrinol (Oxf). 2009; 70(2): 311-321.
151. Palomba S, Falbo A, Orio F, Jr., Zullo F. Effect of preconceptional metformin on abortion risk in polycystic ovary syndrome: a systematic review and meta-analysis of randomized controlled trials. Fertil Steril. 2009; 92(5): 1646-1658.
152. Palomba S, Falbo A, La Sala GB. Effects of metformin in women with polycystic ovary syndrome treated with gonadotrophins for in vitro fertilisation and intracytoplasmic sperm injection cycles: a systematic review and meta-analysis of randomised controlled trials. BJOG. 2013; 120(3): 267-276.
153. Palomba S, Falbo A, La Sala GB. Metformin and gonadotropins for ovulation induction in patients with polycystic ovary syndrome: a systematic review with meta-analysis of randomized controlled trials. Reprod Biol Endocrinol. 2014; 12: 3.
154. Pundir J, Sunkara SK, El-Toukhy T, Khalaf Y. Meta-analysis of GnRH antagonist protocols: do they reduce the risk of OHSS in PCOS? Reprod Biomed Online. 2012; 24(1): 6-22.
155. Pundir J, Psaroudakis D, Savnur P, Bhide P, Sabatini L, Teede H, et al. Inositol treatment of anovulation in women with polycystic ovary syndrome: a meta-analysis of randomised trials. BJOG. 2018; 125(3): 299-308.
156. Raval AD, Hunter T, Stuckey B, Hart RJ. Statins for women with polycystic ovary syndrome not actively trying to conceive. Cochrane Database Syst Rev. 2011; (10): CD008565.
157. Roque M, Tostes AC, Valle M, Sampaio M, Geber S. Letrozole versus clomiphene citrate in polycystic ovary syndrome: systematic review and meta-analysis. Gynecol Endocrinol. 2015; 31(12): 917-921.
158. Siebert TI, Viola MI, Steyn DW, Kruger TF. Is metformin indicated as primary ovulation induction agent in women with PCOS? A systematic review and meta-analysis. Gynecol Obstet Invest. 2012; 73(4): 304-313.
159. Sinawat S, Buppasiri P, Lumbiganon P, Pattanittum P. Long versus short course treatment with metformin and clomiphene citrate for ovulation induction in women with PCOS. Cochrane Database Syst Rev. 2008; (1): CD006226.
160. Siristatidis CS, Vrachnis N, Creatsa M, Maheshwari A, Bhattacharya S. In vitro maturation in subfertile women with polycystic ovarian syndrome undergoing assisted reproduction. Cochrane Database Syst Rev. 2013; (10): CD006606.
161. Siristatidis C, Sergentanis TN, Vogiatzi P, Kanavidis P, Chrelias C, Papantoniou N, et al. In vitro maturation in women with vs. without polycystic ovarian syndrome: a systematic review and meta-analysis. PLoS One. 2015; 10(8): e0134696.
162. Tang T, Lord JM, Norman RJ, Yasmin E, Balen AH. Insulin-sensitising drugs (metformin, rosiglitazone, pioglitazone, D-chiro-inositol) for women with polycystic ovary syndrome, oligo amenorrhoea and subfertility. Cochrane Database Syst Rev. 2012; (5): CD003053.
163. Thakker D, Raval A, Patel I, Walia R. N-acetylcysteine for polycystic ovary syndrome: a systematic review and meta-analysis of randomized controlled clinical trials. Obstet Gynecol Int. 2015; 2015: 1-13.
164. Tso LO, Costello MF, Albuquerque LE, Andriolo RB, Macedo CR. Metformin treatment before and during IVF or ICSI in women with polycystic ovary syndrome. Cochrane Database Syst Rev. 2014; (11): CD006105.
165. Weiss NS, Nahuis M, Bayram N, Mol WB, Van der Veen F, van Wely M. Gonadotrophins for ovulation induction in women with polycystic ovarian syndrome. Cochrane Database Syst Rev. 2015; (9): CD010290.
166. Xiao J, Chen S, Zhang C, Chang S. The effectiveness of metformin ovulation induction treatment in patients with PCOS: a systematic review and meta-analysis. Gynecol Endocrinol. 2012; 28(12): 956-960.
167. Xiao J, Chen S, Zhang C, Chang S. Effectiveness of GnRH antagonist in the treatment of patients with polycystic ovary syndrome undergoing IVF: a systematic review and meta analysis. Gynecol Endocrinol. 2013; 29(3): 187-191.
168. Xingrong T, Shengbing L, Ying C, Chao F, Hua L, Xingping Z, et al. Effect of metformin treatment during pregnancy on women with PCOS: a systematic review and meta-analysis. Clin Invest Med. 2016; 39(4): E120-E31.
169. Zeng XL, Zhang YF, Tian Q, Xue Y, An RF. Effects of metformin on pregnancy outcomes in women with polycystic ovary syndrome: a meta-analysis. Medicine. 2016; 95(36): e4526.
170. Zhang YY, Hou LQ, Zhao TY. Effects of acarbose on polycystic ovary syndrome: a meta-analysis. Exp Clin Endocrinol Diabetes. 2014; 122(6): 373-378.
171. Zhuang J, Wang X, Xu L, Wu T, Kang D. Antidepressants for polycystic ovary syndrome. Cochrane Database Syst Rev. 2013; (5): CD008575.
172. Zhuo Z, Wang A, Yu H. Effect of metformin intervention during pregnancy on the gestational diabetes mellitus in women with polycystic ovary syndrome: a systematic review and meta-analysis. J Diabetes Res. 2014; 2014: 381231.
